# Identification and Expression Analyses of miRNAs from Two Contrasting Flower Color Cultivars of *Canna* by Deep Sequencing

**DOI:** 10.1371/journal.pone.0147499

**Published:** 2016-01-22

**Authors:** Sribash Roy, Abhinandan Mani Tripathi, Amrita Yadav, Parneeta Mishra, Chandra Shekhar Nautiyal

**Affiliations:** 1 Division of Genetics and Molecular Biology, CSIR-National Botanical Research Institute, Rana Pratap Marg, Lucknow, Uttar Pradesh, India; 2 Academy of Scientific and Innovative Research (AcSIR), CSIR-National Botanical Research Institute campus, Rana Pratap Marg, Lucknow, Uttar Pradesh, India; 3 Division of Plant Microbe Interaction, CSIR-National Botanical Research Institute, Lucknow, Uttar Pradesh, India; Nanjing Agricultural University, CHINA

## Abstract

miRNAs are endogenous small RNA (sRNA) that play critical roles in plant development processes. *Canna* is an ornamental plant belonging to family Cannaceae. Here, we report for the first time the identification and differential expression of miRNAs in two contrasting flower color cultivars of *Canna*, Tropical sunrise and Red president. A total of 313 known miRNAs belonging to 78 miRNA families were identified from both the cultivars. Thirty one miRNAs (17 miRNA families) were specific to Tropical sunrise and 43 miRNAs (10 miRNA families) were specific to Red president. Thirty two and 18 putative new miRNAs were identified from Tropical sunrise and Red president, respectively. One hundred and nine miRNAs were differentially expressed in the two cultivars targeting 1343 genes. Among these, 16 miRNAs families targeting60 genes were involved in flower development related traits and five miRNA families targeting five genes were involved in phenyl propanoid and pigment metabolic processes. We further validated the expression analysis of a few miRNA and their target genes by qRT-PCR. Transcription factors were the major miRNA targets identified. Target validation of a few randomly selected miRNAs by RLM-RACE was performed but was successful with only miR162. These findings will help in understanding flower development processes, particularly the color development in *Canna*.

## Introduction

The recent advancement in sequencing technologies has led to the discovery of a wide range of sRNAs from diverse group of organisms. This in turn has helped in understanding the diverse roles of sRNAs in gene regulation during growth and development of an organism. MicroRNAs (miRNAs) are a group of sRNAs, 21–24 nt in length, single stranded and non-coding in nature that are produced from RNA Polymerase II transcripts [1]. It is well established that miRNAs play a critical roles in post-transcriptional gene regulation and control many genes involved in various biological and metabolic processes [2, 3]. There are extensive studies to discover miRNAs and analyze their functions in model plant species, such as *Arabidopsis* and Rice [4]. With the advancement in next generation sequencing technologies, it has become possible to identify species-specific or lowly expressed miRNAs in non-model plants as well [5, 6].

Among other developmental processes, flower development is an important aspect in ornamental plants which largely determines the commercial value of the plants. miRNAs function throughout the flower development processes starting from the earliest floral induction to very late stage like floral organ development, floral organ polarity and defining floral boundaries etc.[7–17]. Some miRNAs regulate members of the florigen and integrator genes involved in flowering, and thus participate in complex genetic networks at floral transition phase [14]. Other miRNAs target and restrict the action of various genes that control different flower-related processes. Several miRNA families are evolutionarily conserved across species. There are at least nine such miRNA families which play critical roles in flower development. These include miR156, miR159, miR160, miR164, miR166/165, miR167, miR169, miR172, and miR319 [14]. These miRNAs mostly regulate flower development by targeting various transcription factors. For example, miR172 regulates floral organ identity and flowering time by translational repression or target cleavage of members of the APETELA2 (AP2) transcription factor genes [18–23]. miR159 is required for normal anther development which it controls through regulating the expression of genes that encode MYB transcription factors [7, 24]. miR156 targets squamosa promoter binding protein-like (SPL) transcription factor gene family to control the transition from the vegetative phase to the floral phase in *Arabidopsis*, rice, and maize [10, 25, 26].

Amongst other floral characteristics, color is an important trait for ornamental flowers. Flower color is mainly due to three classes of pigment: flavonoids, carotenoids and betalains [27]. Among them, a colored class of flavonoids, anthocyanin, confers a diverse range of color from orange to red and violet to blue. Carotenoids and betalains generally yield yellow color. The final color of a flower is determined by a combination of various factors: anthocyanin structures, the pH of the vacuole where anthocyanin localize, coexisting flavonoids (co-pigments) and metal ions etc.[27]. The flavonoids biosynthetic pathway genes including chalcone synthase, transcription factors regulating these genes and P450s relevant to flower color have also been well studied [28]. Besides others, SPL and R2R3-MYB transcription factors negatively regulate flavonoid biosynthesis [28, 29]. More recently the role of miRNAs in regulating these genes have been reported [5, 28]. Kim et al. (2012) in analyzing miRNAs from different rose cultivars observed enrichment of five miRNAs (miR171, miR166i, miR159e, miR845, and miR396e) in white cultivar of rose [5]. They suggested that these miRNAs may negatively regulate target genes to prevent accumulation of carotenoids or flavonoids resulting in white flowers. However, they could not validate any target genes and thus were not able to confirm the miRNA-directed cleavage of target genes involved in color determination in rose. But they validated miR156 and miR159 as the target of SPL and R2R3-MYB transcription factors, respectively [5]. Similarly, sequencing and degradome analysis of Apple miRNAs revealed large number of R2R3-MYB transcription factors primarily involved in anthocyanin biosynthesis were targets of miR858 [30]. It was further shown that a majority of those MYBs were co-targets of miR828 [31, 32]. These results indicate miRNAs play critical roles in regulation of color development in plant tissues.

However, in spite of rapid advancement in next generation sequencing technologies and our understanding on molecular mechanisms of flower development regulated by miRNAs in model as well as non-model plant species [5, 6], there are only a few studies on miRNAs from ornamental plants [5]. One of the reason may be due to the non availability of genomic resources from these group of plants. Because, it is necessary to predict and construct hairpin precursors of potential miRNAs by using neighbouring genomic sequences of the mapped sRNAs to distinguish high quality miRNAs from other sRNAs [33, 34]. Therefore, miRNA identification and deciphering their probable roles in flower and color development in ornamental flowers will greatly enhance our knowledge in the field.

*Canna* is a perennial flowering ornamental plant. It is reported to be originated in central and South America. There are 8–10 species of *Canna* which are widely used as ornamental plant [35]. It grows abundantly in the humid tropical and subtropical regions throughout the world. The flowers are born singly or in pairs and arranged into larger branched clusters at the tips of the flowering stems. Each flower appears to have five petals but these are actually modified stamens (staminodes) [36]. Since its first hybridization in 19^th^ century, a large number of hybrids have evolved by crossing of different *Canna* species. These hybrids often grouped under the names of *C*. *x generalis* L.H. Bailey and*C*. *x orchioides* L.H. Baley [35]. There are over 1000 known cultivars ranging from less than 0.75 to 2.4 meter in height and variable in colors from creams, yellow, orange and red.

In this study, we report for the first time deep sequencing of miRNA from *Canna* cultivars, the only member of the family Cannaceae belonging to the order Zingiberales. To the best of our knowledge, except *Musa acuminata* no other plant genome sequence is known from this taxonomic order. Therefore, identification of miRNAs from this plant species is challenging. Our aim was to identify the conserved miRNAs and their differential expression in two contrasting flower color cultivars of *Canna* which might play important role in flower and color development. We also predicted a few novel miRNAs from both the cultivars. Overall,we identified 313 known and 50 putative novel miRNAs from both the cultivars. One hundred and nine miRNAs were differentially expressed between the two cultivars. The differentially expressed and the putative novel miRNAs may provide insight into the molecular mechanisms of flower color as well as other development processes in *Canna*.

## Materials and Methods

### Plant Samples

**Fig 1:**
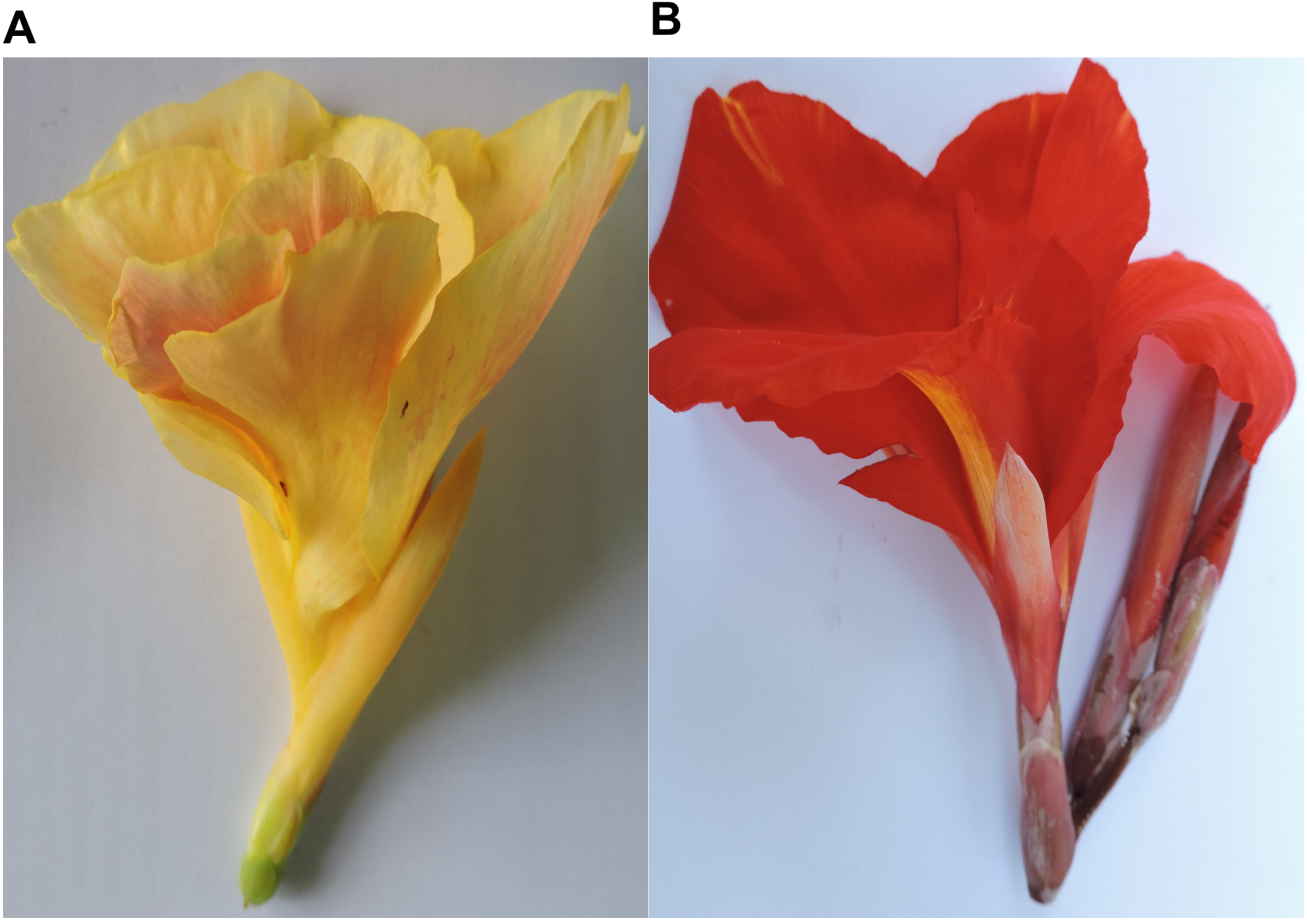
Two *Canna* cultivars having contrasting flower color (A) Tropical sunrise (B) Red president.

We have chosen two contrasting flower color *Canna* cultivars, the Tropical sunrise (TS) and Red president (RP) for deep sequencing of miRNA. The TS *Canna* have pale yellow flower with large glossy pointed dark green leaves, whereas the RP have large red flowers and lush green leaves “Fig 1”. The cultivars are maintained at the Institute's research field for various other research activities. Randomly selected first fully opened flowers from each of the two cultivars were collected from the field grown plants during mid of July, 2013. The flower tissues were immediately frozen in liquid nitrogen and brought to the laboratory for RNA isolation. Fresh tissues were also collected for phytochemical estimation.

### Quantification of Flavonoids and Carotenoids

Total flavonoids, carotenoids (carotene and xanthophylls) and anthocyanins were extracted from the staminodes of the two *Canna* cultivars. One gram of tissue was taken as starting material for extraction of the phytochemicals. Total flavonoids was extracted using AlCl_3_ method [37] and absorbance was measured at 440 nm using spectrophotometer (SpectraMax Plus 384, USA). The anthocyanins were extracted from staminodes in acidic methanol (1% HCl, w/v) for 48 hrs in dark with a ratio of 10 ml buffer to 1 gm sample following Mancinelli et al (1934) [38]. The absorbance of anthocyanin was measured at 530 nm. Carotenoids were extracted following the method of Chen and Yang (1992) [39]. The absorbance of carotene and xanthophylls was measured at 436 nm and 474 nm, respectively. All the absorbance measurements were taken with five technical and three biological replicates for each of the two cultivars.

### sRNA libraries preparation and sequencing

Total RNA was extracted from staminodes tissue using Trizole-LS reagent (Invitrogen, USA) according to the manufacturer’s instructions. The integrity of RNA was checked by running on Agarose gel as well as using Agilent Bioanalyzer (Agilent Technologies, U.S.A). The RNA was run on 15% Tris-Borate-EDTA (TBE) urea denturating polyacrylamide gel and the 20 to 30 nt small RNA fraction was extracted and eluted. Next, the sRNA molecules were ligated to 5' adaptor and a 3' adaptor sequentially and then converted to cDNA by RT-PCR. A single cDNA library was prepared for each cultivar. The resulting libraries were sequenced at Center for Cellular and Molecular Platform (C-CAMP, Bangalore, India) using Illumina Hiseq platform. The raw data of both the libraries has been submitted to NCBI under accession no SRA310114.

### Analysis of sRNAs

The raw reads from the two libraries were first cleaned by removing 5' and 3' adaptors and low quality reads (< 30 Q values)by using FASTAX-Toolkit. These clean reads were subjected to BLASTn search against Sanger RNA database (Rfam) (http://www.sanger.ac.uk/software/Rfam). The reads matching with other RNAs, including rRNA, tRNA, snRNA and snoRNA were excluded and remaining reads were designated as filtered reads. The filtered reads were submitted to the UEA sRNA toolkit-Plants version, miRProf pipeline (http://srna-workbench.cmp.uea.ac.uk/tools/analysis-tools/mirprof/) [40] at default parameters which aligned the sequences to miRBase 19.0 (http://www.miRBase.org) [41] and provided raw and normalized read counts of known miRNAs.

Since no other genomic information is available on *Canna*, we used *Musa acuminata*, taxonomically the nearest taxa to *Canna* with known genome sequence to predict miRNA precursors. Secondary structure was predicted using miRCat (http://srna-workbench.cmp.uea.ac.uk/tools/analysis-tools/mircat/) [40] at default parameters. The predicted secondary structure were analyzed manually by using following parameters 1) The miRNA and miRNA* are derived from opposite stem arms such that they form a duplex with two nucleotide 3' overhangs, 2) base-pairing between the miRNA and miRNA* is extensive such that there are typically four or fewer mismatches, 3) The frequency of asymmetric bulges is one or none and size of the bulges is no more than two nucleotide within miRNA/miRNA* duplex [42]. These miRNAs were further submitted to miRProf pipeline. The putative miRNAs that were mapped on previously reported miRNAs were designated as known and unmapped as putative novel miRNAs. The detailed pipeline of deep sequencing data analysis is shown in “S1 Fig”.

### Determination of differentially expressed miRNAs

All the read counts were normalized to reads per million (RPM). If original miRNA expression in a library was zero, the normalized expression was adjusted to 0.001 according to a previous report [43]. Log2 fold change in miRNA expression were calculated between the two libraries only when read counts were more than 10 in any one of the libraries. A 2x2 contingency table was used to perform Pearson’s chi-square test of significance between the two libraries [44]. We considered expression to be significant when fold change was ≥ 1 and P value was ≤ 0.05 for a particular comparison.

### Validation of known and putative novel miRNAs and their target genes by qRT-PCR

The expression level of known miRNAs were checked by using poly (A) method [45]. Five hundred nanograms of total RNA were poly (A) tailed and reverse transcribed using NCode miRNA first-strand cDNA synthesis kit (Invitrogen, USA) according to user’s manual. For novel miRNAs, cDNAs were synthesised from stem loop RT-PCR [46] by using SuperScript^®^ III Reverse Transcriptase (Invitrogen, USA) for each of the four miRNAs following manufacturer's instructions. For the miRNA target identification, cDNAs were prepared by using GoScript^™^ Reverse Transcription System (Promega, USA). To check the expression level of the known and the novel miRNAs, the synthesized cDNAs were amplified using the miRNA specific forward primers and universal reverse primers [47]. To check the expression level of the target genes of the known miRNAs, cDNAs were amplified using gene specific primers. The primer sequences are listed in “S1 Table”. qRT-PCR was performed with DyNAmo Flash SYBR Green (Thermo) with cycling conditions: denaturation at 95°C for 10 min, followed by 40 cycles of denaturation at 95°C for 20 s, annealing and extension together at 60°C for 60s. The amplification reaction was performed using ABI7300 real-time PCR system (Applied Biosystem). All the reactions were performed with two biological and three technical replicates. 5.8S and 18S rRNA were taken as an endogenous control for miRNAs and their target genes, respectively. The expression data of samples were normalized by using expression value of 5.8S and 18S rRNA and relative expression was calculated using 2^−ΔΔCt^ method. The fold change values were determined using 2^−ΔΔCt^ method [48].

### Prediction of miRNA targets and GO analysis

The *Musa acuminata* transcripts were downloaded from the database (http://banana-genome.cirad.fr/content/download-dh-pahang) for target prediction. The psRNA target program (http://plantgrn.noble.org/psRNATarget/) was used for target prediction at default parameters. The GO terms of the miRNA targets were annotated according to their biological role, molecular function and cellular component by using the online GO term analysis tool (http://bioinfo.cau.edu.cn/agriGO/analysis.php).

### Target validation by modified 5' RLM-RACE

To validate putative targets of a few selected miRNAs viz. miR156, miR159 and miR162 RLM 5′-RACE was carried out using First Choice RLM-RACE kit (Ambion,USA) following user's manual. Briefly, total RNA was isolated from flower tissue and adapter was ligated. The ligated RNA was used for cDNA synthesis using random hexamer. The PCR amplification was performed using the adapter specific outer primer and gene specific outer primers. Nested PCR amplification were performed using the adapter specific inner primer and gene specific inner primers “S1 Table”. The PCR product was separated on 2% agarose gel and distinct bands of the appropriate size of the miRNA target genes were eluted. The eluted PCR product was cloned in pGEM-T easy vector (Promega,USA) and at least ten individual clones were sequenced for each target.

## Results

### Flavonoids and Carotenoids contents of TS and RP flower tissues

**Fig 2:**
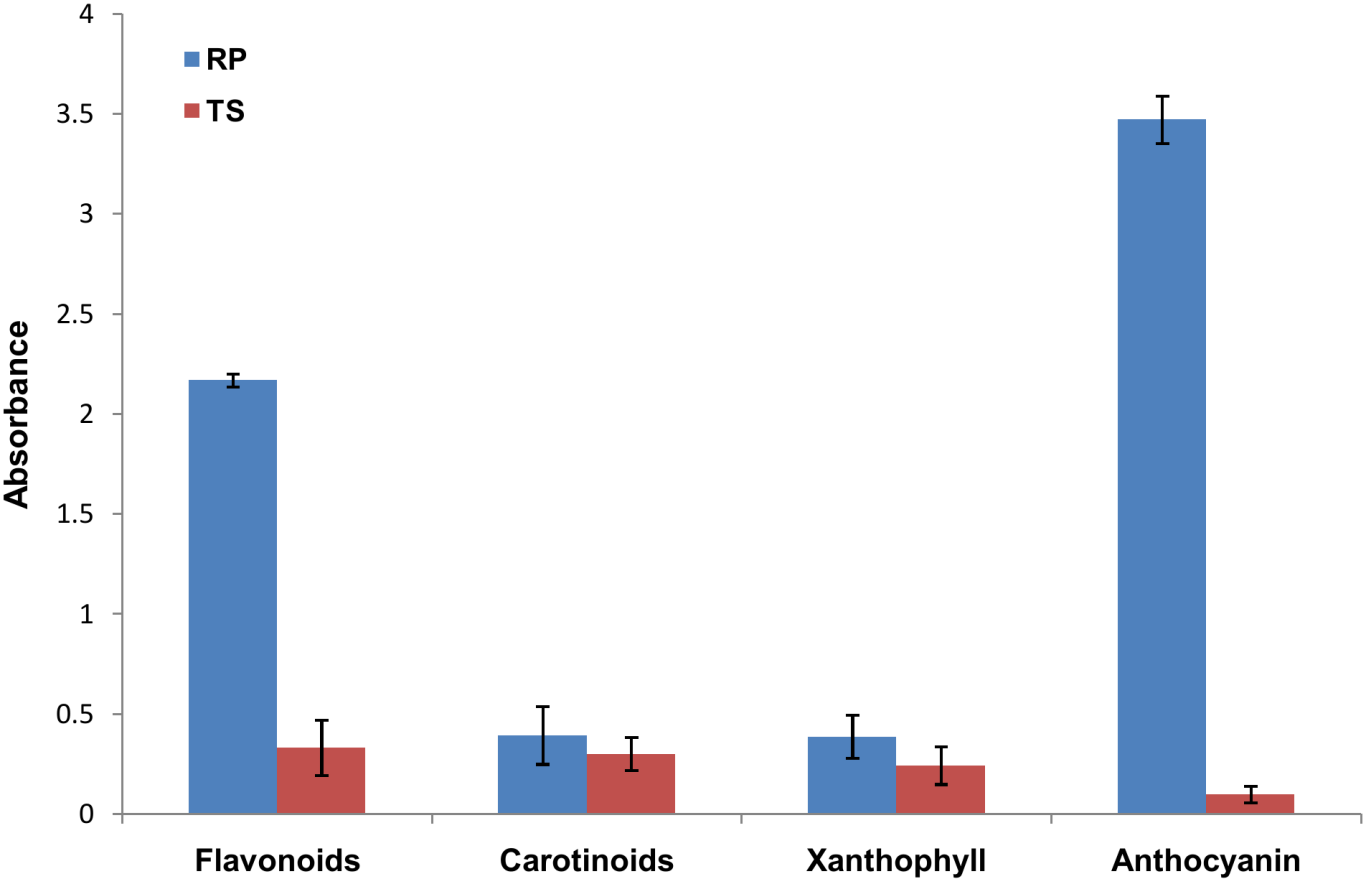
Flavonoids, Carotenoids, Xanthophyll and Anthocyanin content of TS and RP. All the measurements were taken in three biological and five technical replicates. Error bars represent standard deviation of biological and technical replicates.

We estimated total flavonoids as well as anthocyanin and carotenoids from the flower tissue of the two cultivars. As shown in the “Fig 2”, the total flavonoids and anthocyanin content was much higher in RP than TS. On the other hand carotenoids and xanthophyll content of RP was very less as compared to the total flavonoids and anthocyanin. Carotenoids and xanthophyll content was less in TS than RP. These indicate flavonoids and anthocyanin are the major source of color pigment in *Canna*.

### Sequencing of sRNA from *Canna* flower

**Fig 3:**
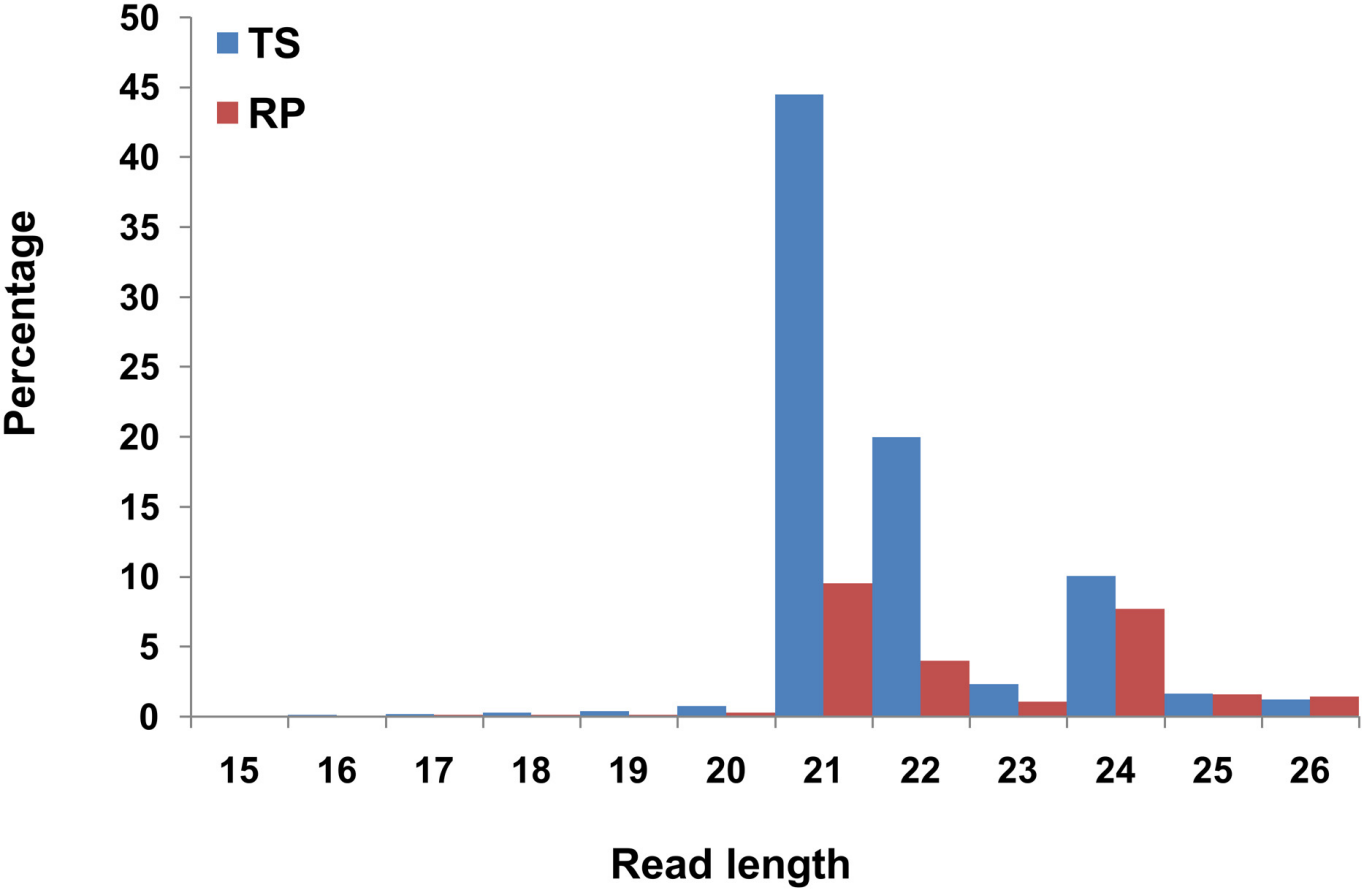
Length distribution of sRNAs in two libraries, TS and RP. Two sRNA libraries were prepared from staminode tissue of two contrasting flower cultivars of *Canna*. Size distribution of the sRNA libraries show that the 21 nt size miRNAs were dominating ones in both the libraries.

Two sRNA libraries from staminode tissuesof TS and RP were constructed. Sequencing of these sRNA libraries generated ~28.0 million and ~ 18.7 million absolute reads from TS and RP, respectively. The raw reads were processed to remove the adaptor sequences, low quality sequences and other small RNAs including tRNA, rRNA, snRNA and snoRNAs. Other small RNAs were filtered out by BLASTn against Rfam database. This resulted approximately 21.3 million and 10.89 million filtered reads from TS and RP, respectively “Table 1”. The size distribution of the raw reads is depicted in “Fig 3”. The majority of these RNA reads were of 21, 22 and 24 nt in length, whose production relied on DCL4, DCL2 and DCL3, respectively [49, 50]. Most abundant reads were of 21 nt length, the characteristic of canonical miRNAs. Filtered reads were mapped with miRBase19.0. 67.40% of the filtered reads of TS and 28.24% filtered reads of RP mapped with the miRBase 19.0 sequences “Table 1”. To identify the precursors of the known and the novel miRNAs, the filtered reads were further mapped to *Musa acuminata* genome (http://banana-genome.cirad.fr). This resulted in mapping of 42.9% reads of TS and 56.9% reads of RP to *Musa acuminata* genome "Table 1".

**Table 1:** Categorization and abundance of sRNA reads from TS and RP.

	absolute reads		unique reads	
	TS	RP	TS	RP
Data Processing				
Total sequencing reads	28040652	18475931	4594182	3324234
rRNA	6331713	6039143	3931	2773
tRNA	380941	1535094	9072	4964
snoRNA	7163	3384	2178	1303
snRNA	328	547	83	63
Filtered reads	21320507	10897763	4578918	3315131
Reads mapped to miRBase19.0	14371676	3077840	3290	1703
reads mapped to miRNA*	114914	4523	535	165
sRNA align to banana genome	12029439 (42.9%)	10512804 (56.9%)		

### Identification of known miRNAs in *Canna*

**Fig 4:**
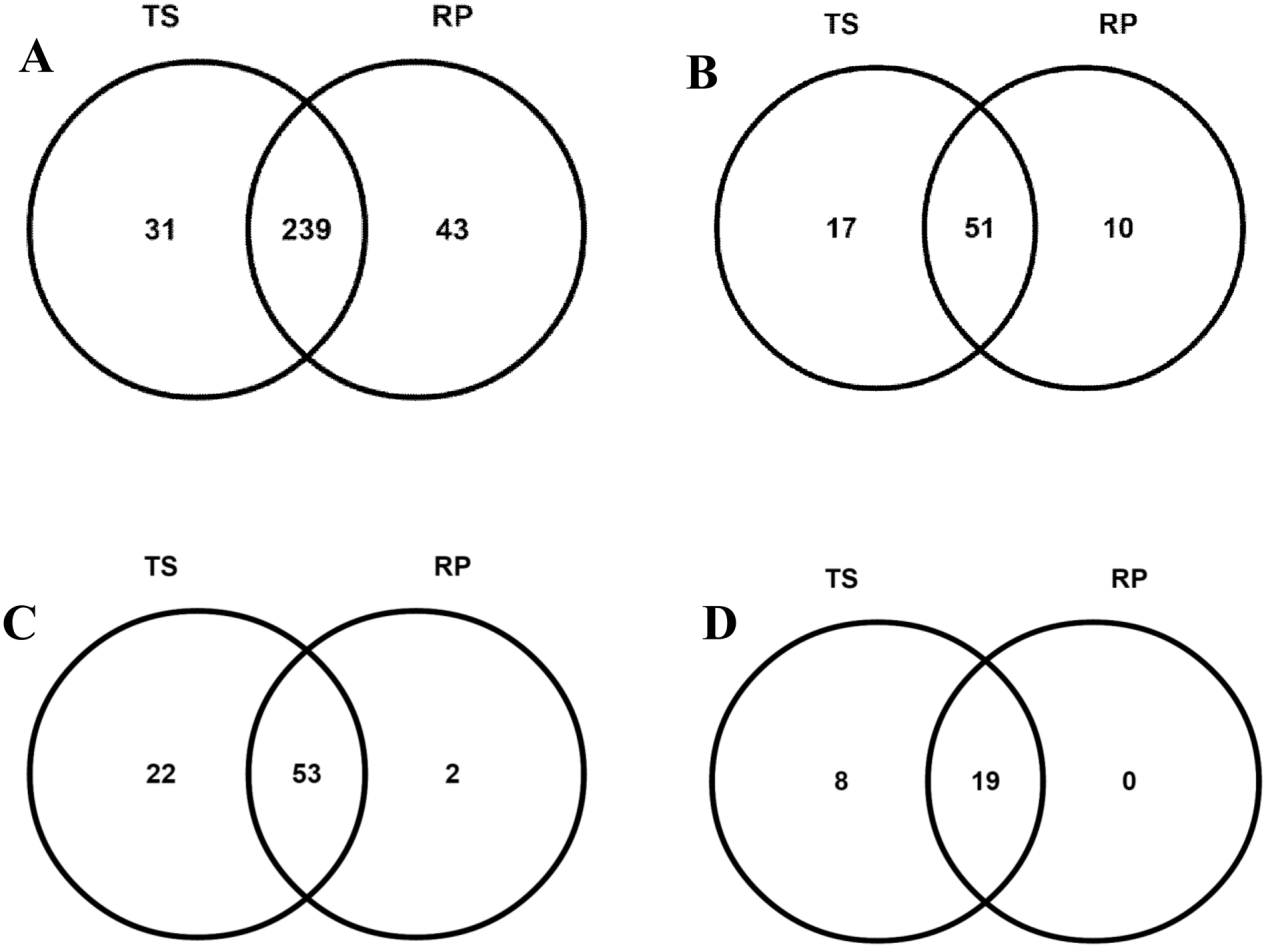
Venn diagrams of conserved and unique miRNAs between TS and RP. (A) Total conserved and unique miRNAs between TS and RP, (B) Conserved and unique miRNA families between TS and RP, (C) Total conserved and unique miRNA* between TS and RP, (D) Conserved and unique miRNA* families between TS and RP.

In order to identify the known miRNAs (both conserved and non-conserved), the filtered reads were mapped with miRBase 19.0. The small RNA sequences that matched with miRBase database were identified as the known miRNAs in *Canna*. A total of 313 miRNAs belonging to 78 miRNA families were identified from both the libraries. There were 271 miRNAs belonging to 68 miRNA families in TS and 282 miRNAs belonging to 61 miRNA families in RP “S2 Table”. Among these identified miRNAs, 239 miRNAs (51 families) were common in both TS and RP. Thirty one miRNAs (17 miRNA families) were specific to TS and 43 miRNAs (10 miRNA families) were specific to RP “Fig 4A and 4B”.

**Fig 5:**
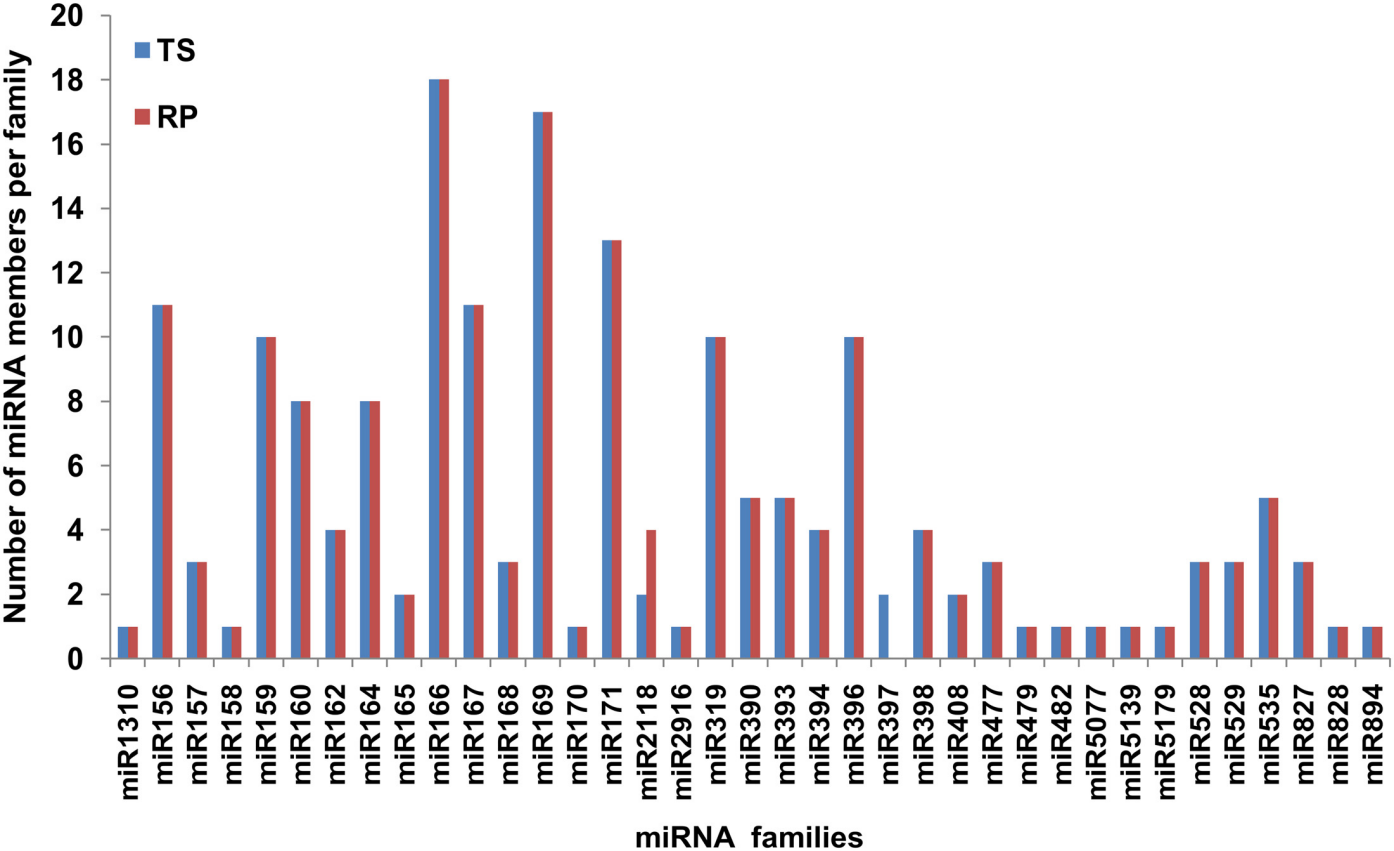
Number of miRNAs members in each family identified from TS and RP by deep sequencing.

Among the known miRNAs, most of the conserved miRNA families had higher read abundance in both the libraries, e.g. miR166 had more than one hundred thousand reads. The non-conserved families had read abundance of one to four thousands “S2 Table”. Further, as reported earlier the conserved miRNA families had higher number of members per family as compared to the non-conserved miRNA [51]. For example, miRNA families miR156, miR159, miR166, miR167, miR169, miR171, miR319 and miR396 were highly conserved and exhibited higher number of members, whereas several known but non-conserved miRNAs families*viz*. miR408, miR477, miR479 and miR529 had one to three members per miRNAs family “Fig 5”.

We further analyzed the miRNAs* and their families in both the libraries. A total of 75 and 55 miRNAs* belonging to 27 and 19 miRNA* families were identified in TS and RP, respectively “S3 Table”. Further analysis revealed that 53 miRNA* belonging to 19 miRNA* families were common in both the libraries. Twenty two miRNA* belong to eight miRNA* families were specific to TS, whereas only two miRNA* with no specific miRNA* families were found in RP “Fig 4C and 4D”. Interestingly, some miRNAs* have more read counts than their corresponding miRNAs “S4 Table”.

### Identification and validation of novel miRNAs in *Canna*

**Fig 6:**
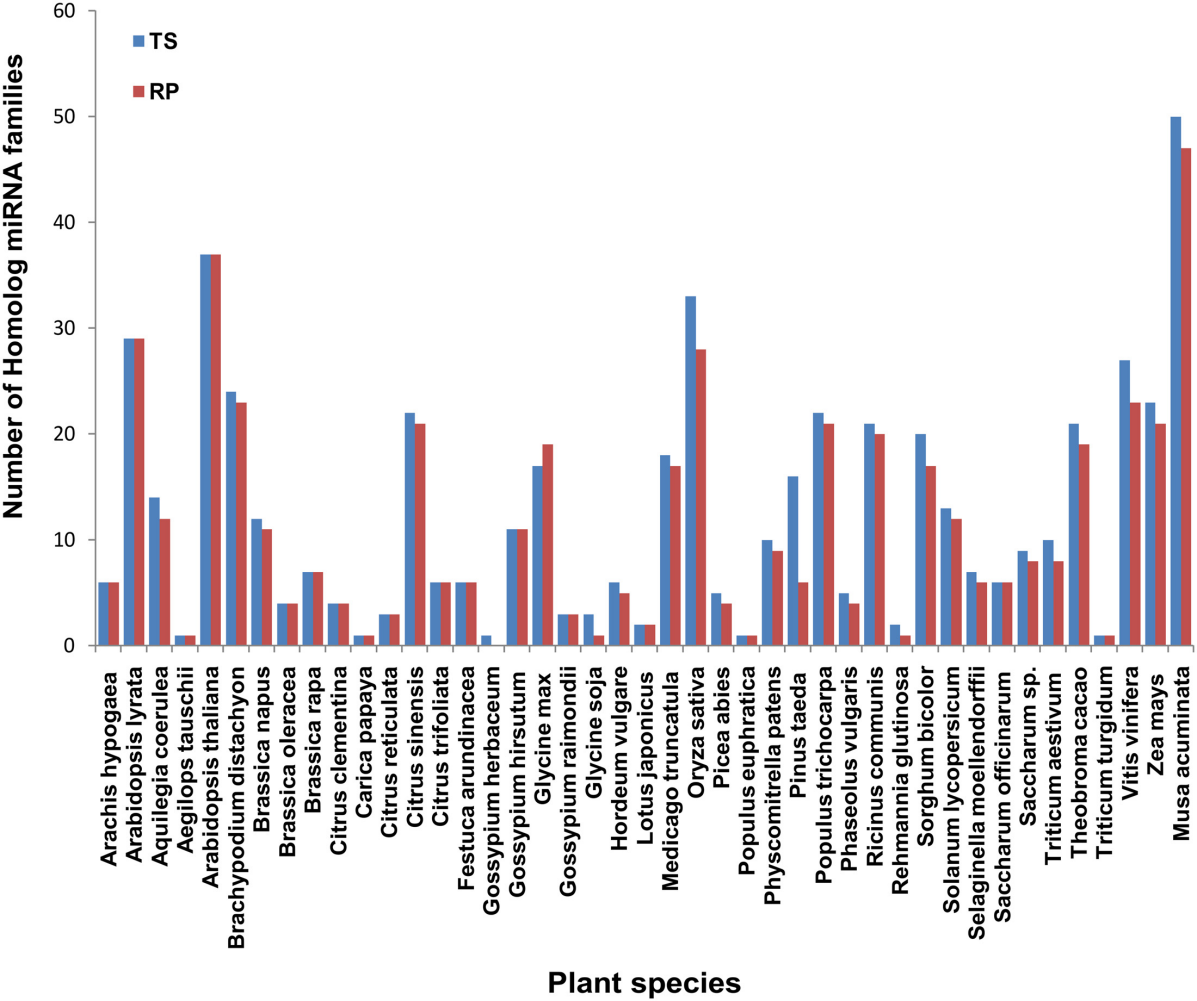
The homology of identified miRNAs with other plant species. Values on Y axis indicate the number of homological miRNA families between *Canna* and other plant species.

In order to identify the novel miRNAs, we mapped the raw reads with *Musa acuminata* genome [52]. Further, homology search of all the known miRNAs using miRBase 19.0 and reported *Musa acuminata* miRNAs [53] revealed that *Canna* miRNAs exhibited the highest number of homology with *Musa*, followed by *Arabidopsis thaliana* and *Oryza sativa* “Fig 6”. Therefore, we chose to map *Canna* sRNA reads with *Musa acuminata*. 42.90% and 56.90% reads of TS and RP, respectively mapped with *Musa* genome “Table 1”. Based on predicted secondary structures and the standard criteria of plant miRNAs [42], 205 and 160 miRNAs precursors and their corresponding mature miRNAs were predicted from TS and RP, respectively. These predicted miRNAs were again mapped with the plant miRNAs reported in miRBase 19.0. There were 32 and 18 miRNAs from TS and RP, respectively which did not map with known miRNAs and we predicted these miRNAs as putative novel miRNAs. Among these, 10 miRNAs were common in both TS and RP with three miRNAs showing their corresponding miRNA* sequences “S5 Table”, “S2 Fig”.Out of these putative novel miRNAs, four randomly selected miRNAs were further validated by stem loop RT-PCR “S3 Fig”.

### Expression pattern of known miRNAs in TS and RP

**Fig 7:**
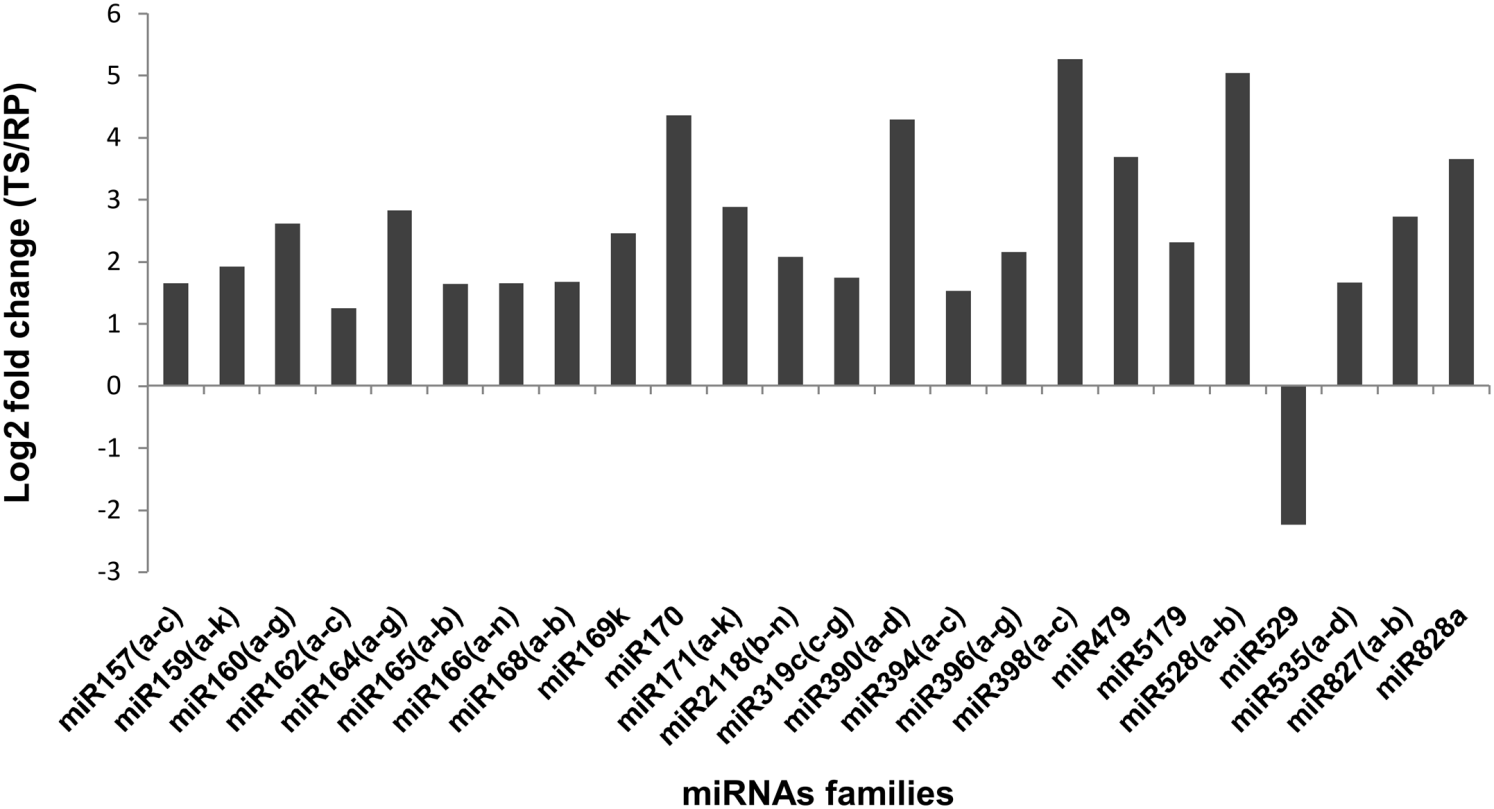
Differentially expressed miRNA families between TS and RP.

Differential expression of miRNAs was analyzed as described in materials and methods. Expression analysis showed 109 miRNAs belonging to 22 families were differentially expressed in the two cultivars “S6 Table”. Among the families, miR398 and miR528 expressed at five fold and miR170, miR390, miR479 and miR828 expressed at four fold higher in TS as compared to RP “Fig 7”. Expression of only miR529 was higher in RP as compared to TP (~ 2.2 fold). Further, miR397, miR397b and miR2118e, miR2118r expressed only in TS and RP, respectively “S6 Table”. These results indicate that ~ 34% miRNAs which were differentially expressed in two cultivars might play an important role in flower development.

### Validation of miRNA expression by qRT-PCR

**Fig 8:**
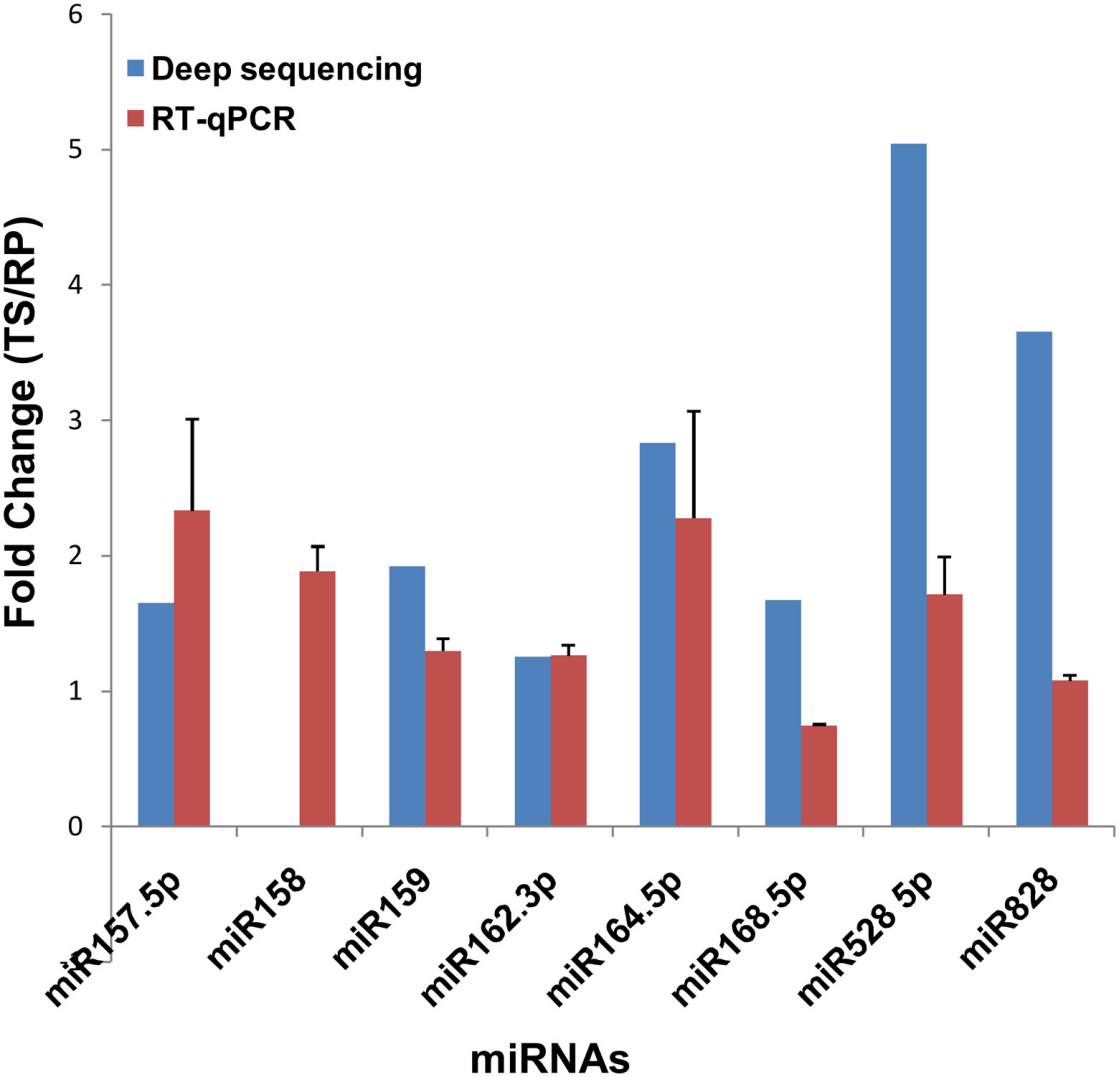
Comparison of the miRNA expression levels determined by deep sequencing and qRT-PCR. Blue and red colors indicate the fold change obtained by deep sequencing and qRT-PCR, respectively. The error bars indicate the standard deviation obtained from biological and technical replicates.

Out of 22 differentially expressed miRNA families as indicated in deep sequencing, eight families were randomly chosen for validation of expression pattern using qRT-PCR. As shown in “Fig 8”, seven miRNA families exhibited similar expression level in both deep sequencing and qRT-PCR analysis.

### Identification and enrichment analysis of miRNAs target genes

The biological role of miRNAs is better understood by the functions of their target genes. It is also reported that the conserved miRNAs may regulate the conserved targets [42]. Therefore, with the aim of better understanding the biological role of the conserved and the novel miRNAs, we searched for putative target genes by using a plant sRNA target analysis tool, psRNATarget [54] at default parameters. The details of the target gene ID, nature of inhibition, unpaired energy are shown in “S7 Table”. A total of 1343 targets were identified for 109 significantly differentially expressed miRNAs. Transcription factors were the major miRNA targets identified. Among these transcription factors, squamosa promoter binding proteins like (SPL), MYB-domain transcription factors (MYB), ARF-auxin responsive factors (ARF), NAC-domain transcription factors (NAC), CUC-cup shaped cotyledon (CUC), homeodomain leucine zipper transcription factors (HD-ZIP) and TCP domain transcription factors (TCP) were the important ones “S7 Table”. Other miRNA targets were growth regulating factors, laccase, transporter activity, ATPase domain containing protein, kinase proteins and chaperone proteins etc. The targets for the putative novel miRNAs were mainly transporter genes, amino transferses, heat shock proteins and protein kinase “S8 Table”.

### Experimental validation of known miRNA targets

**Fig 9:**
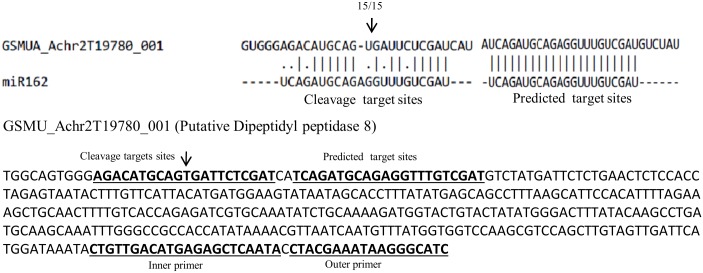
Detection of cleavage site through RLM-RACE. 5' RLM RACE was used to map the cleavage sites. The partial mRNA sequence from the target genes were aligned with the miRNA. The arrow indicates the cleavage site, and the number above the arrow denotes the frequency of the sequenced clones.

Cleavage site of predicted target of conserved miRNAs in *Canna* were validated by using a modified form of 5' RLM-RACE. Though we selected a few miRNAs for target validation, however we were successful in validating the target of only miR162. As reported earlier, miR162 had two putative targets viz. dipeptidyl peptidase 8 [55] and endoribonuclease dicer homolog 1 [56]with expected E value less than two. The latter target was experimentally validated in earlier report [56]. However, in *Canna* only Dipeptidyl peptidase 8 was identified as the target of miR162. The Dipeptidyl peptidase 8 has two adjacent target sites for miR162that forms miRNA/target duplex. Out of these two sites, one was perfect complementary to miR162 and other one had one nucleotide indel and five nuclotides mismatch in miRNA/target duplex region. In RLM-RACE experiment, only the latter traget was identified “Fig 9”. While this type of miRNA target site was not very common for most of the validated plant miRNA targets but has been observed in another case [57].

**Fig 10:**
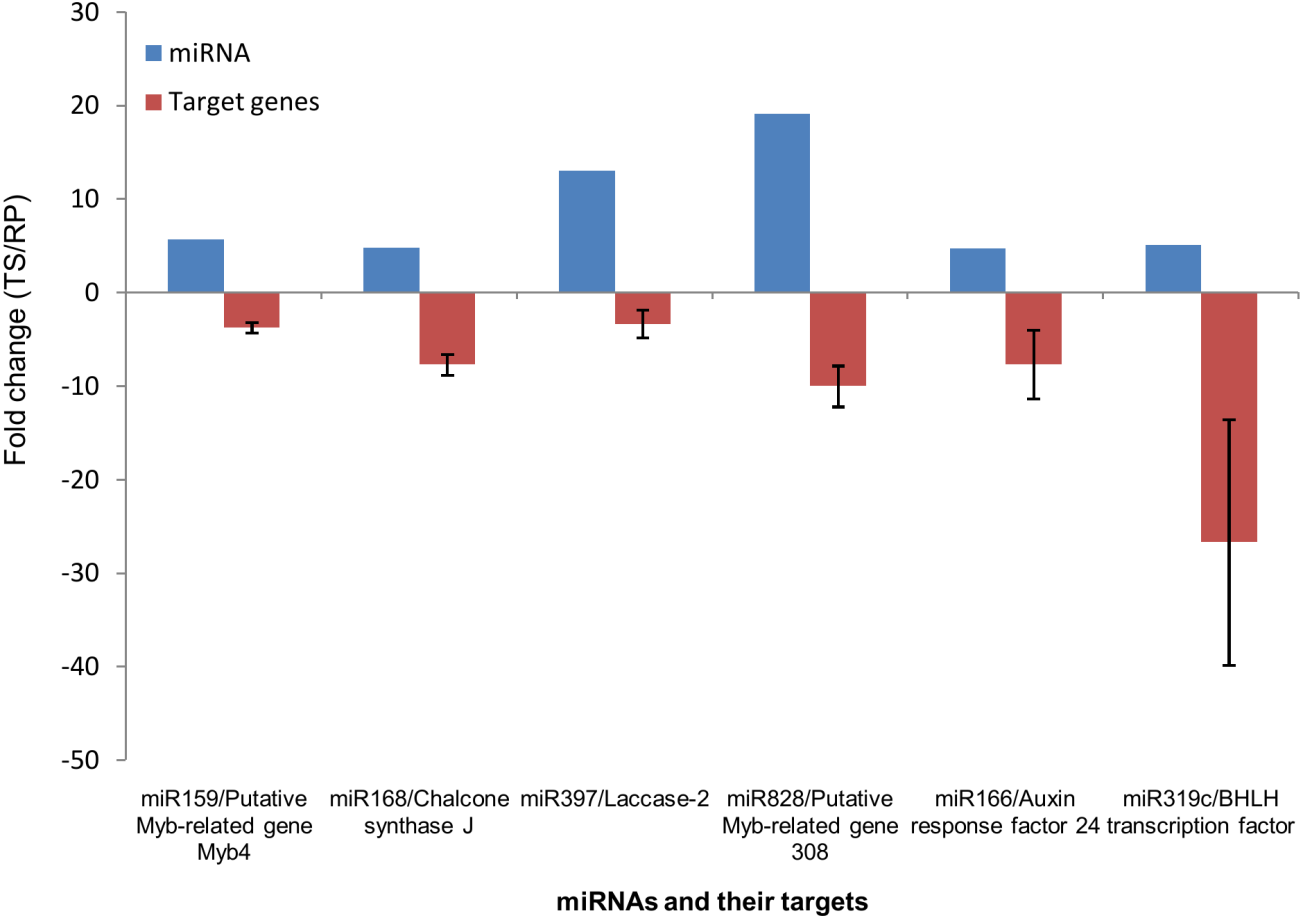
Correlation of miRNAs and their target genes expression in TS and RP. The target genes of selected miRNAs were analyzed by qRT-PCR. The expression level of these target genes (red) were compared with the expression level of corresponding miRNAs (blue) estimated from sequencing data. Error bars indicate mean ± standard deviation of three biological and three technical replicates.

Further, we analyzed the expression level of the predicted miRNA target genes which were involved in pigment and flower development processes by qRT-PCR. In TS library, the expression of MYB related genes, chalcone synthase and laccase which are involved in pigment biosynthesis were down regulated, where as the expression of miRNAs viz. miR159, miR828, miR168 and miR397 which target these genes were up regulated. While, miR159 and miR828 known to target different MYB related genes [32], miR397 is known to target laccase genes [58]. On the other hand, in RP, the expression level of these genes and the miRNAs targeting these genes were up and down regulated, respectively “Fig 10”. Further, on the basis of miRNA expression data, target analysis and validation experiment by qRT-PCR, we predict miR168, which targets ARGONAUTE1 (AGO1) [59] may also target chalcone synthase gene in *Canna* flowers. These results indicate the involvement of these miRNAs in regulation of pigment related genes in *Canna* flowers.

### Functional annotation of differentially expressed miRNAs

**Fig 11:**
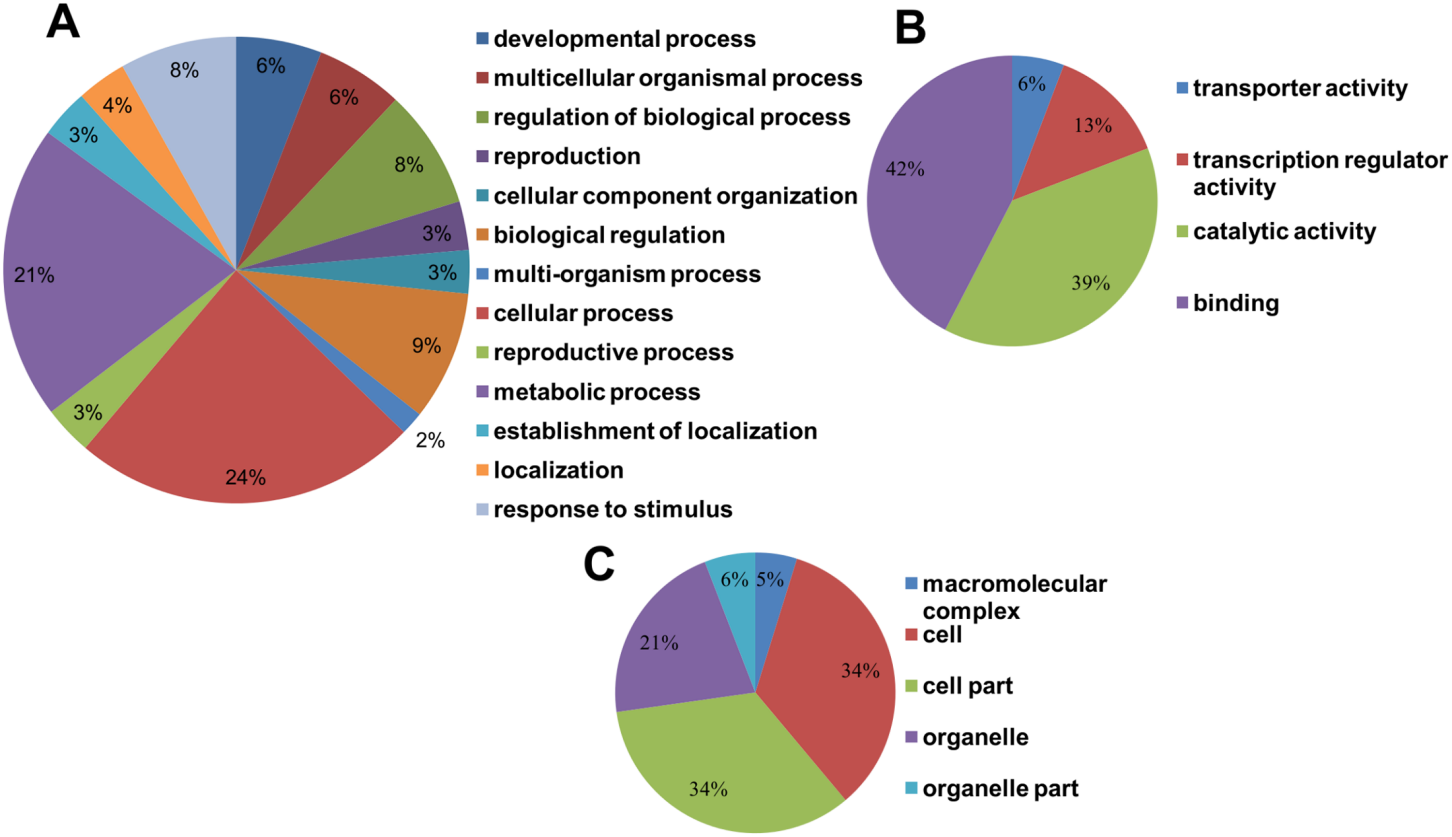
Gene ontology enrichment analysis of the predicted targets of *Canna* miRNAs. Categorization of miRNA-targets genes were performed according to the(A) Biological Processes, (B) Molecular function and (C) Cellular component.

For better understanding the role of identified miRNA targets, we performed AgriGO analysis,a promising method for uncovering the miRNA-gene regulatory network. A total of 1343 identified targets of 109 differentially expressed miRNAs gave 633 GO terms. These were further categorized in Biological process (398 terms), Cellular component (84 terms) and Molecular function (151 terms). Under biological processes, major target genes were involved in cellular process (24%), metabolic process (21%), biological regulation (9%) and developmental process (6%) “Fig 11A”. In molecular component major target genes were involved in binding processes (42%), catalytic activity (39%) and transcription regulator activity (13%) “Fig 11B”. The cellular component included major three targets which were involved in cell part (34%), cell (34%) and organelle function and developments (21%) “Fig 11C”. We were interested to see the major targets involved in flower development related traits. Our analysis revealed that 16 miRNAs which targeted 60 genes were involved in flower development related traits such as fertilization, floral whorl development, fruit development and sexual reproduction “S9 Table”. Since the cultivars were contrasting in terms of flower colors, we further analyzed the targets for secondary metabolite pathways including pigment biosynthesis. This revealed five miRNAs families which targets five genes involved in phenyl propanoid metabolic process and pigment metabolic process “S9 Table”. Expression of all these miRNAs that are related to flower development and pigment biosynthesis were higher in TS than RP, except for miR529 “Fig 7”.

## Discussion

*Canna* is one of the important ornamental flowering plant because of its attractive and vibrant flower colors. Yet,it's use in cut flower trade is limited because of its very short shelf life. The petals drupe within a few hours of plugging. However, it has great potential to be used as cut flower if flower related traits including shelf life can be enhanced. In spite of having great values, there is no report on molecular studies especially related to flower development in *Canna*. Understanding the molecular mechanisms in shaping flower ornamental quality in *Canna* will provide great value in breeding of different *Canna* cultivars.

A large number of miRNAs have been reported from different plant species [59]. Most of these studies are based on plant species whose genome is well characterized [60–62]. More recently focus has been made to identify new miRNAs from plant species with unknown genome sequence [5, 6]. miRNAs are known to regulate almost all developmental processes in organisms. Identification of new miRNAs and analysis of differential expression of miRNAs in two contrasting cultivars will provide further insight into their role in plant developmental processes. A total of 313 miRNAs (78 miRNA families) and corresponding 130 miRNA* (56 miRNA* families) were identified from both the cultivars. Due to lack of genome sequences or ESTs of *Canna*, miRNA precursors were predicted based on sRNAs mapped with *Musa acuminata* genome, which is taxonomically nearest taxa having known genome sequence. The mapping of sRNA to cross species is possible as miRNAs are highly conserved between species.This also maximizes the screening of novel miRNAs in plants for which genome is not available [5, 12, 63]. This approach has generally been followed for miRNA profiling in organisms with unknown genome sequences [5, 63]. However, this may under/ over-estimate the actual numbers of miRNAs due to evolutionarily loss/gain of genomic region and expression pattern, especially when one compares with taxonomically distant clad, as in the present study. Nevertheless, this approach has provided fair miRNA profiling in cross-species studies [5, 63, 64]. Identification of large numbers of miRNAs in *Canna* based on mapping of very distant taxa suggests that *Canna* shares many conserved miRNAs between ornamental and fruit bearing plants. Similarly,a very high degree of conservation of miRNAs was also observed between Rosaceae and Strawberry [5]. We further detected a large numbers of miRNA* familes indicating existence of authentic miRNAs. The abundance of miRNA* is usually much lower than that of their corresponding miRNAs. This is because of rapid degradation of miRNA* after their complementary miRNA sequences are selected from the miRNA/miRNA* duplex and loaded into the AGO protein [65, 66]. However, we observed a few miRNAs* sequences whose read counts were more than their corresponding miRNAs. Similar observation wasalso reported in other study [67]. Further, in both the libraries the reads of conserved miRNAs were larger as compared to non-conserved miRNAs. For example, miR166 have more than one hundred thousand reads. It is reported that conserved miRNAs generally have high reads abundance than non conserved miRNAs [63].

Discovery of novel miRNAs from species with unknown genomic information is more challenging. However, based on mapping with nearest genome sequence and subsequent prediction of secondary structures using the standard criteria of plant miRNAs [42], we identified 32 and 18 miRNAs as putative novel miRNAs in TS and RP, respectively. Predicted target genes for these miRNAs were related to transporter gene, heat shock protein etc. Further characterization and functional validation of these miRNAs and their target genes may provide insight into the *Canna* specific flower development process. Because of very contrasting flower color and differences in some other morphological traits between the two cultivars, we hypothesized that there would be high degree of differential expression of a range of miRNAs. As expected, we observed as many as 109 miRNAs which were differentially expressed in the two cultivars. Amongst 109 differentially expressed miRNAs, expression of only miR 529was higher in RP cultivar where as all other miRNAs expressed at 1–5 fold higher in TS as compared to RP cultivar.

Since we were interested to explore flower related traits and their regulation, we focused on major targets of miRNAs involved in this process. After assessing the GO terms by enrichment analysis we found most of the conserved miRNAs were involved in development of various flower parts. Functional annotation showed that16 miRNAs which targeted 60 genes were involved in flower related traits. Most of these predicted targets that are involved in *Canna* flower development have already been confirmed in model plants [14]. For example, it is reported that miR164 defines the flower boundary by targeting no apical meristem protein [13, 68]. The floral boundary, which also refers to the overall size of the flower was smaller in TS than RP. This is consistent with expression of miR164 which was higher in TS as compared to RP (further validated by qRT-PCR).

Flower color is one of the important characteristic of any flowering ornamental plant. Therefore, it is important to understand the color development in individual plant species for better utilization of genotypes by breeders. Flower colors are determined by accumulation of secondary metabolites such as flavonoids, carotenoids, and betalains [27, 69]. The biochemical estimation showed that flavonoids were the major source of color development in *Canna*. The low level of carotenoids in TS as compared to RP, but comparable amount of carotenoids in both the cultivars indicated carotenoids may not be the major source of pigments in floral color development of *Canna*. A previous study also suggested higher flavonoids content in red flower variety of *Canna* than yellow one [70]. We examined the miRNAs and their targets which were involved in the color development pathways. Five miRNAs were identified which were differentially expressed between the two cultivars. Amongst these, miR159 and miR828 target different types of R2R3-MYB transcription factors. These transcription factors form an (MYB-bHLH-WDR) initiation complex and are involved in the early steps of flavonoid biosynthesis pathway [71]. Thus miR159 and miR828 plays important role in regulation of flavonoid biosynthesis pathway. The expression of these miRNAs was 2–3 folds higher in TS cultivar as compared to RP cultivar. The higher level of expression in TS might down regulate the early steps of flavonoid biosynthesis pathway and hence inhibit pigment biosynthesis. Similarly, expression of miR168 which target chalcone synthase (CHS) was also higher in TS as compared to RP. Chalcone synthase (CHS) is involved in the first step of flavonoid pathway. On the other hand miR535 and miR397 target the intermediate enzymes, coumarate CoA ligase and laccase which are intermediate enzymes in flavonoid pathway. All these miRNAs were expressed at higher level in TS as compared to RP. The validation of some of these target gene expression by qRT-PCR further reconfirmed our findings about their role in flower color develpment in *Canna*. However, flower color development is known to be regulated by combinatorial mechanisms and may not be an act of straight regulation by single or a few miRNAs and their targets. Therefore, it is difficult to elucidate the molecular mechanism of miRNA-directed regulation of color determination in *Canna*. More particularly when no other genomic resources are available from the species. Further, target validation of a few selected miRNAs including those involved in pigment biosynthesis genes largely failed in our experiment. We validated target of miR162 that encodes an enzyme dipeptidyl peptidase 8. This target carries two miRNA target sites, one with a high level of complementarity and the other one is a duplex with six nucleotides mismatches. The identified new target demonstrates that miRNA/target duplex need not to be a strictly complementary to their target site. Though this type of unconventional traget sites is uncommon but was reported in ealier study [57]. Nevertheless, miRNA profiling of these two contrasting *Canna* cultivars will certainly provide potential clue about *Canna* flower color development.

## Conclusions

Understanding the mechanism of processes associated with flower development in different flowering plants is an important aspect of miRNA regulated processes. Here, we reported differential expression of miRNAs in two cultivars of *Canna*. Our analysis showed that there were 31 and 43 miRNAs which were specifically expressed in TS and RP cultivars. These miRNAs might play a crucial role in cultivar specific flower development processes. The integrated analysis of miRNAs and their targets suggested that differential expression of miRNAs in two cultivars might be responsible in regulation of morphological as well as pigment biosynthesis pathways. The putative new miRNAs might provide further clue in gene regulation of flower development processes in ornamental plants, particularly in *Canna*.

## Supporting Information

S1 Fig miRNA analysis pipeline. (PDF)

S2 Fig Secondary structure of 50 putative novel *Canna* specific miRNAs and miRNA*. (PDF)

S3 Fig Quantitative analyses of novel miRNAs by stem-loop real-time PCR. (TIF)

S1 Table List of primers sequences that were used in this study. (XLSX)

S2 Table Known miRNAs and their families. (XLSX)

S3 Table miRNAs having star sequences. (XLSX)

S4 Table Differentially expressed miRNAs and miRNAs* in TS and RP. (XLSX)

S5 Table Putative new miRNAs predicted in this study by miRCat algorithms. (XLSX)

S6 Table Significantly differentially expressed miRNAs between TS and RP cultivars. (XLSX)

S7 Table Predicted targets of known miRNAs. (XLSX)

S8 Table Predicted targets of putative novel miRNAs. (XLSX)

S9 Table miRNAs involved in flower and color development. (XLSX)
