# Supplementary figures and images for: Identification and Expression Analyses of miRNAs from Two Contrasting Flower Color Cultivars of Canna by Deep Sequencing
Source: PLoS One. 2016 Jan 22;11(1):e0147499. doi: 10.1371/journal.pone.0147499 (PMC4723037; doi:10.1371/journal.pone.0147499)

S1 Figure miRNA analysis pipeline

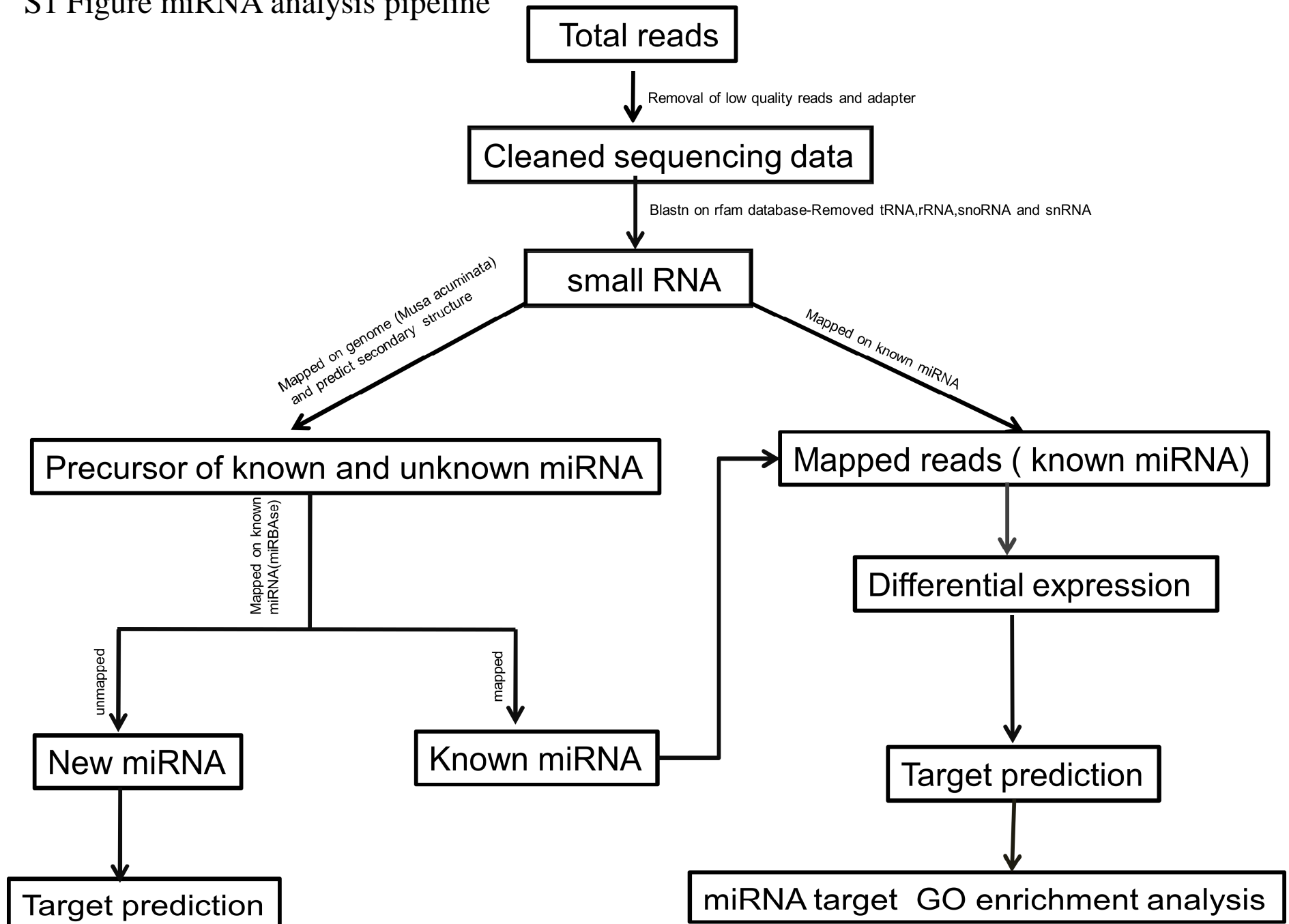

Supplement: S1 Fig — (PDF) [file pone.0147499.s001.pdf]

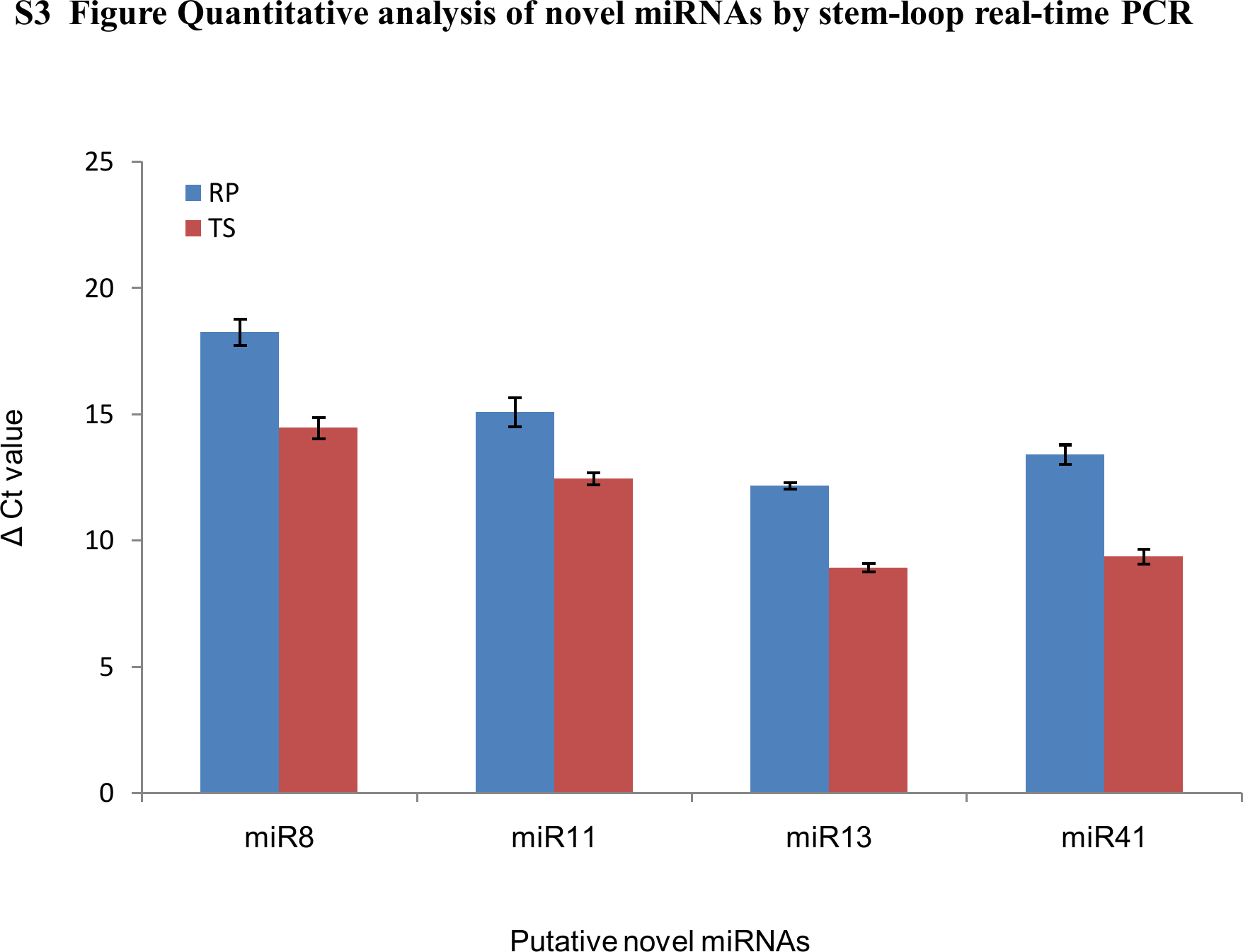

Supplement: S3 Fig — (TIF) [file pone.0147499.s003.tif]
