# Supplementary material for: Identification and Expression Analyses of miRNAs from Two Contrasting Flower Color Cultivars of Canna by Deep Sequencing
Source: PLoS One. 2016 Jan 22;11(1):e0147499. doi: 10.1371/journal.pone.0147499 (PMC4723037; doi:10.1371/journal.pone.0147499)
Supplement: S2 Fig — (PDF) [file pone.0147499.s002.pdf]

S2 Figure Secondary structures of 50 putative novel *Canna* specific miRNAs and miRNAs* counterparts

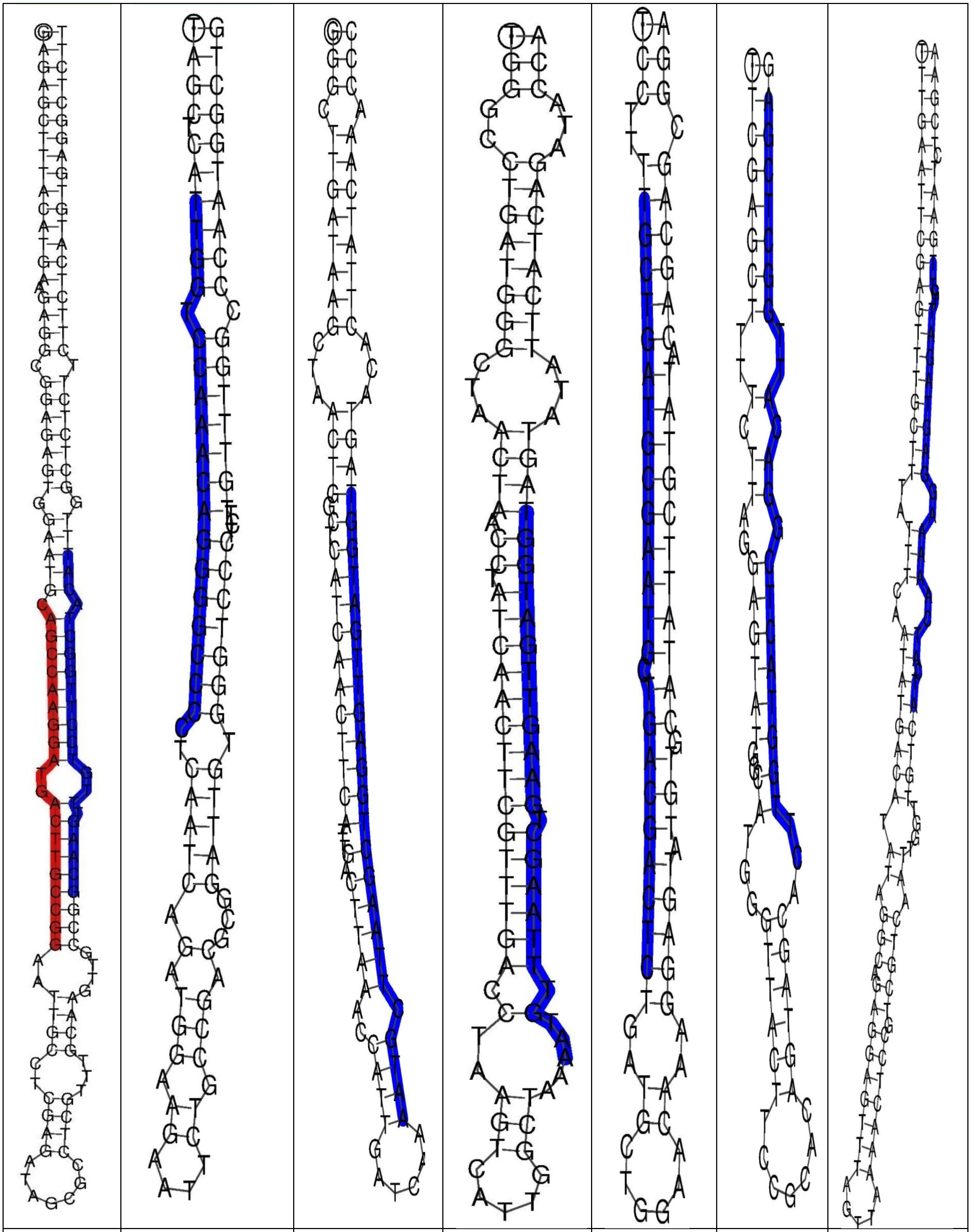

miRNA1	miRNA2	miRNA3	miRNA4	miRNA5	miRNA6	miRNA7

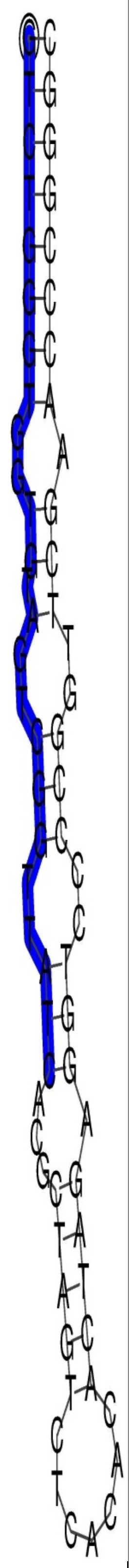	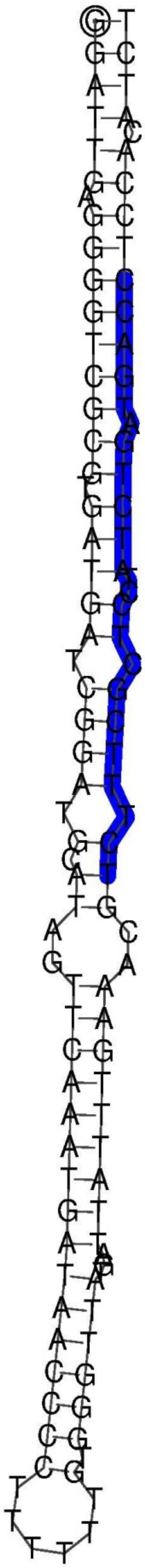	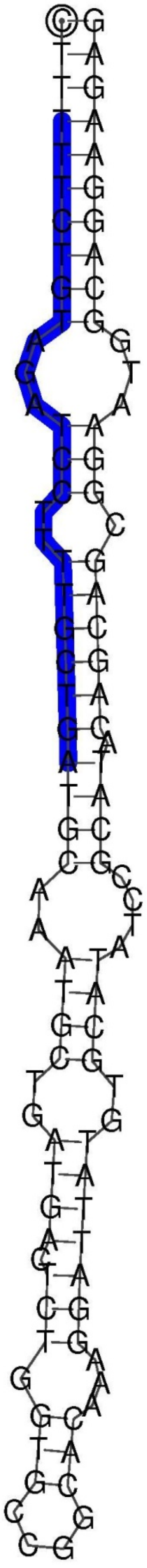	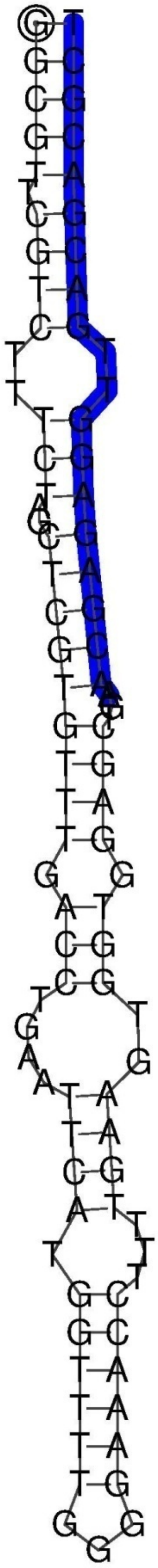	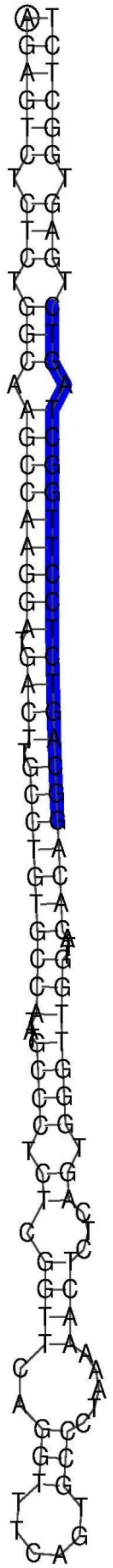	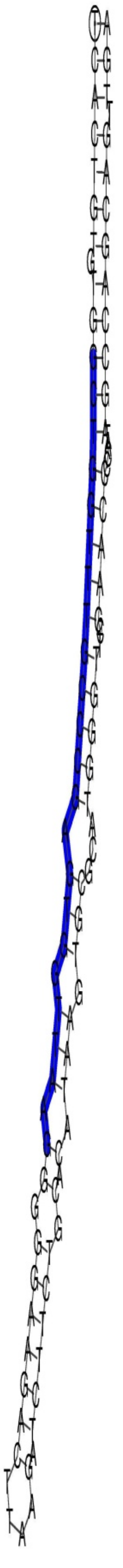	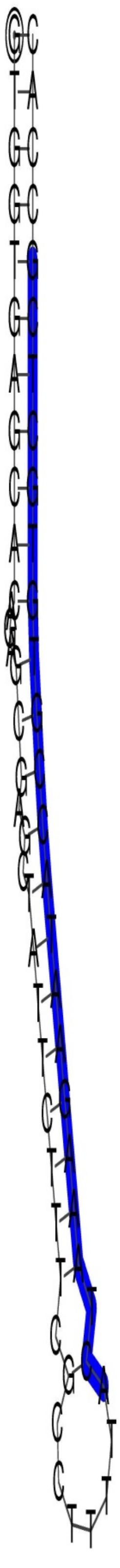
miRNA8	miRNA9	miRNA10	miRNA11	miRNA12	miRNA13	miRNA14

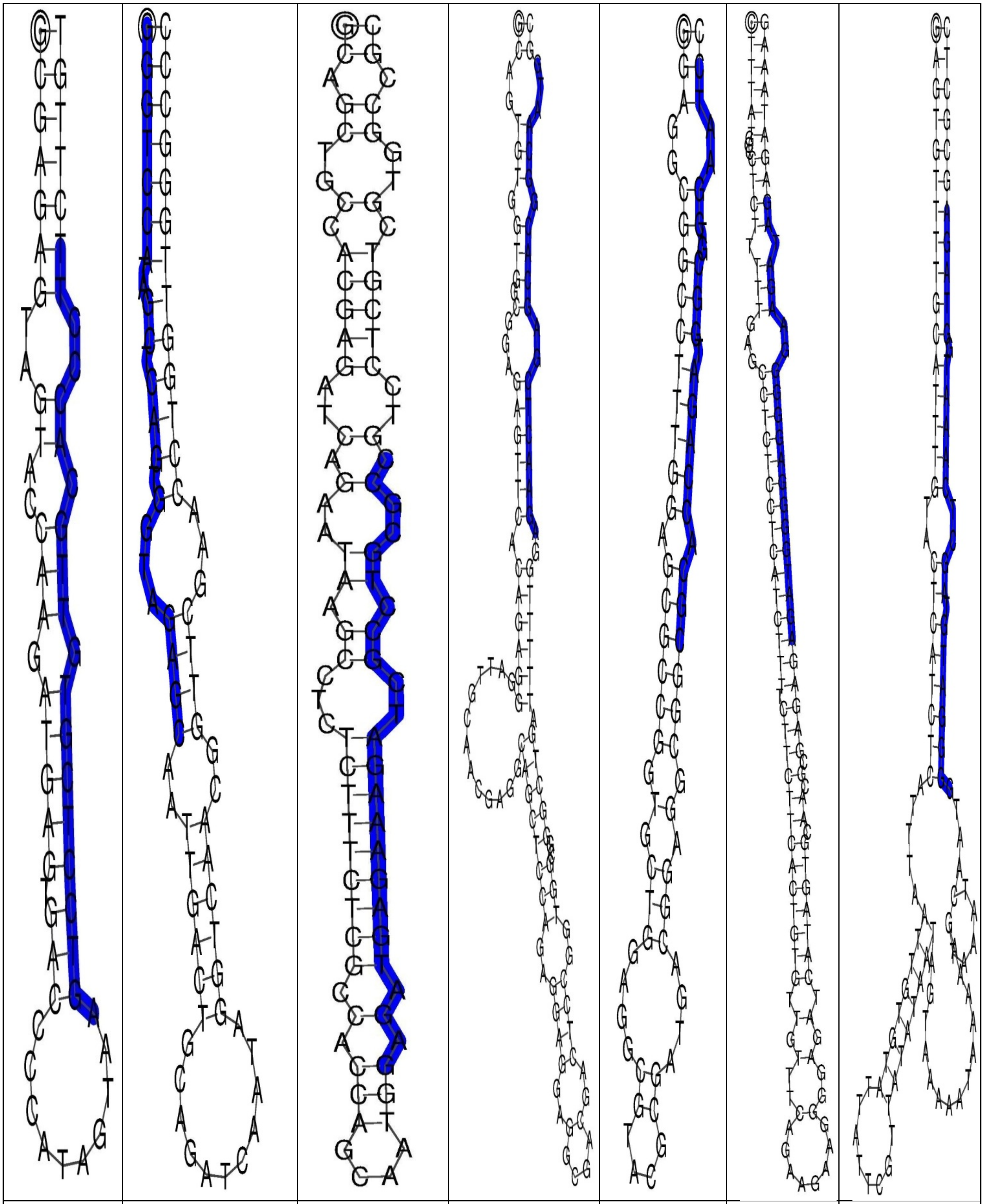

miRNA15	miRNA16	miRNA17	miRNA18	miRNA19	miRNA20	miRNA21

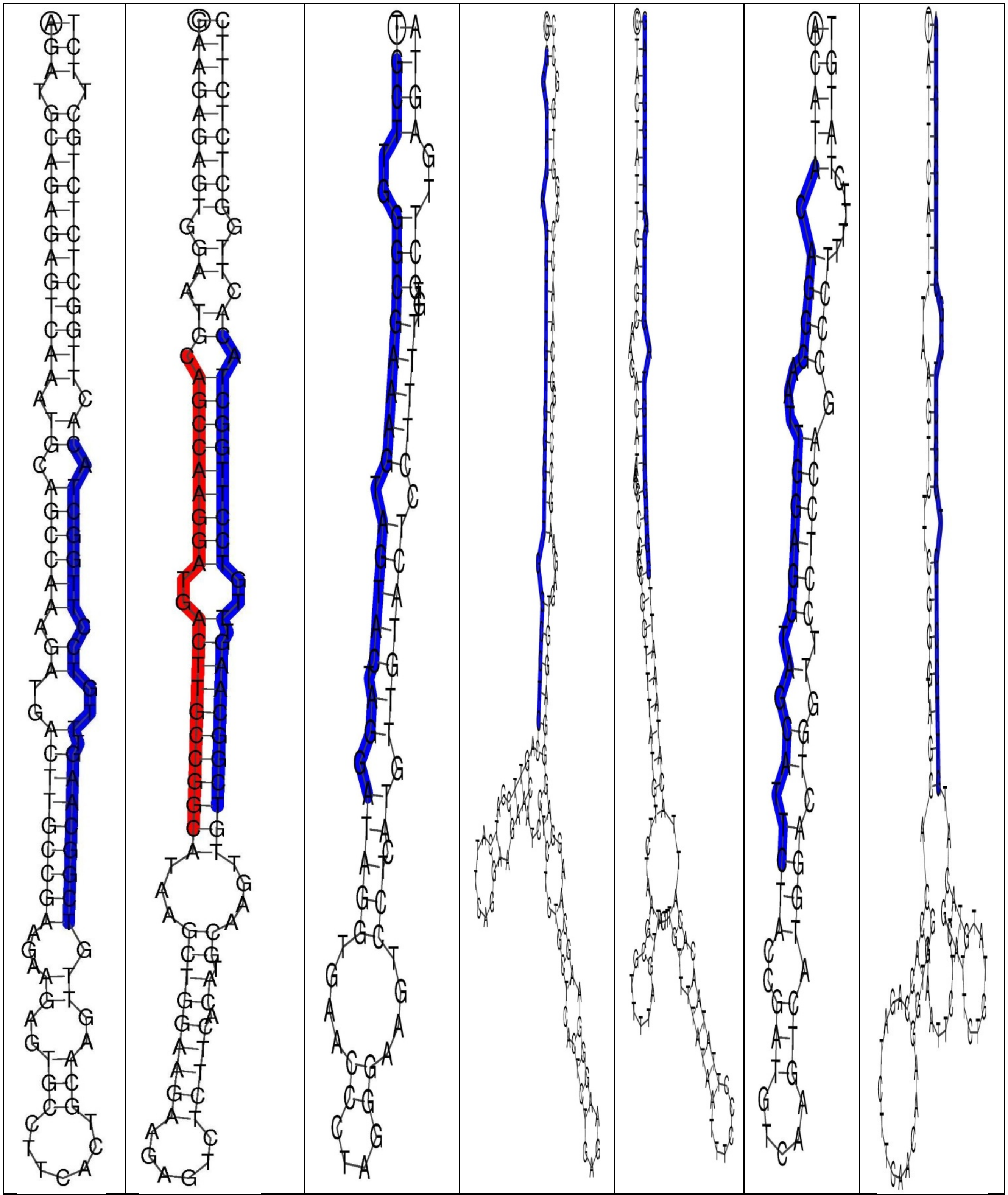

miRNA22	miRNA23	miRNA24	miRNA25	miRNA26	miRNA27	miRNA28

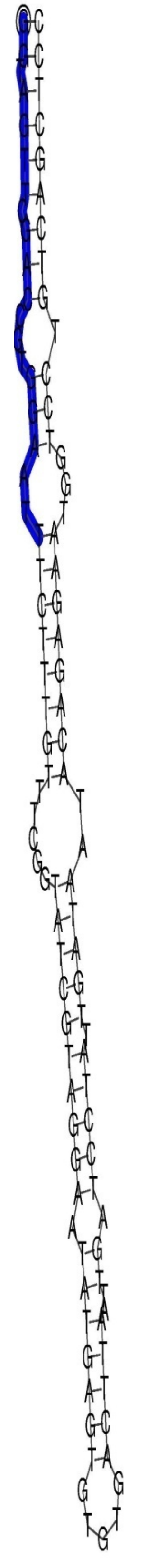	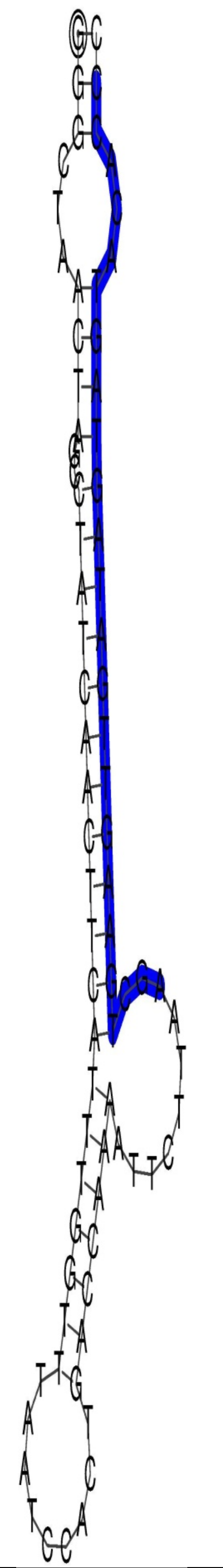	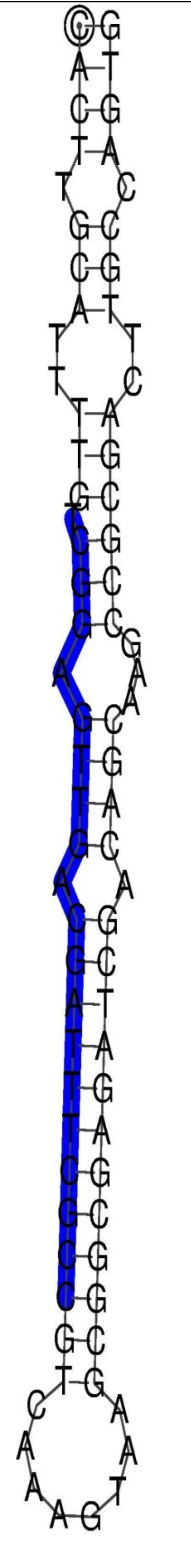	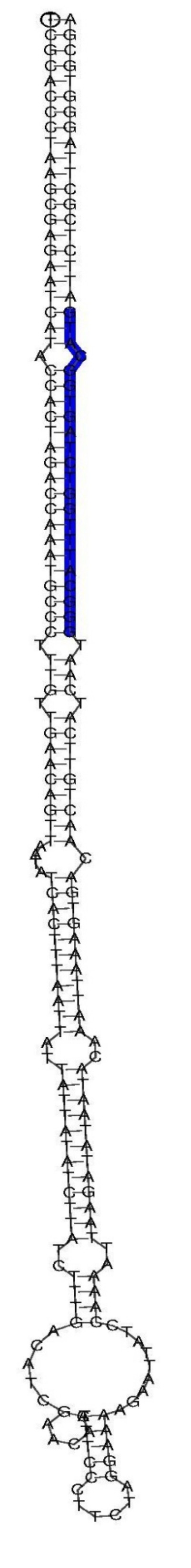	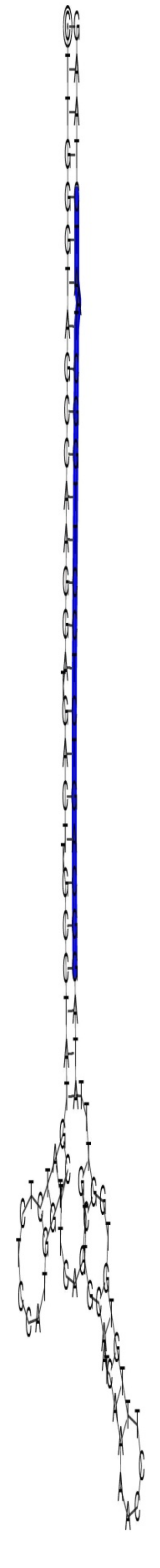	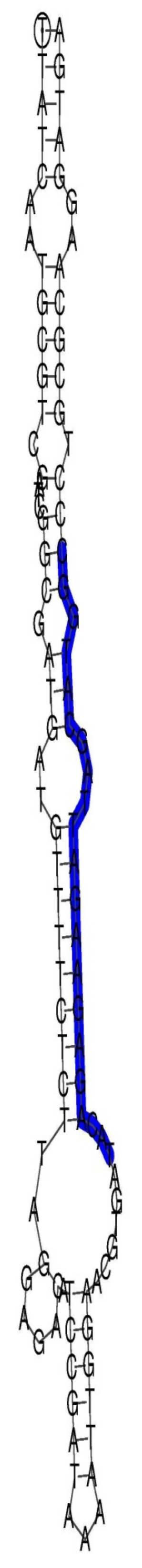	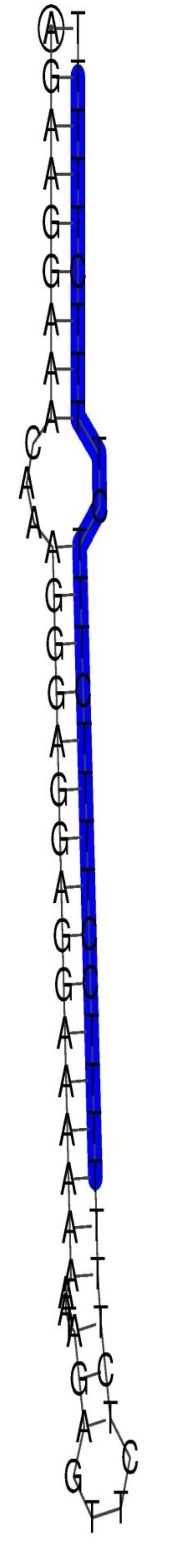
miRNA29	miRNA30	miRNA31	miRNA32	miRNA33	miRNA34	miRNA35

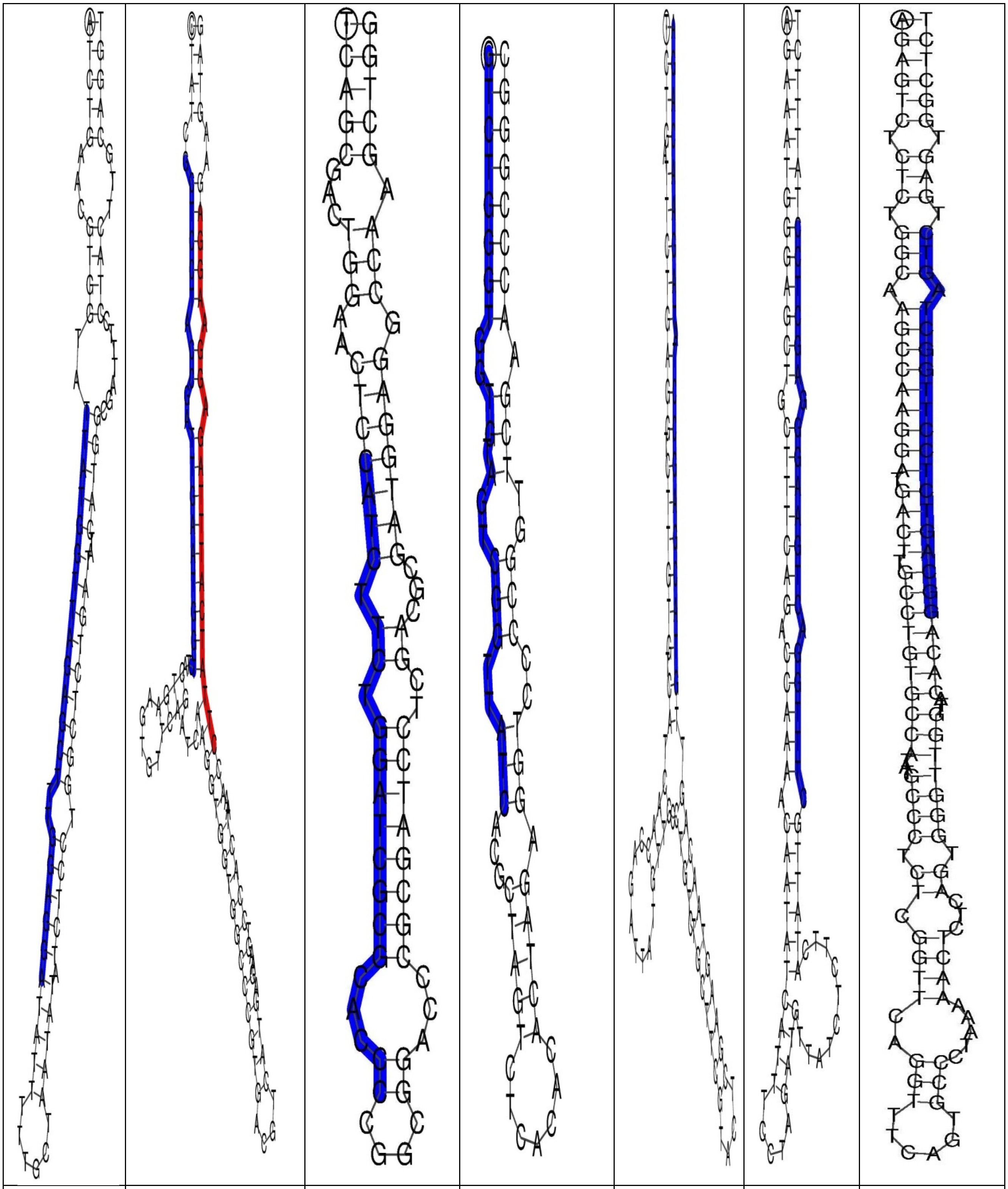

miRNA36	miRNA37	miRNA38	miRNA39	miRNA40	miRNA41	miRNA42

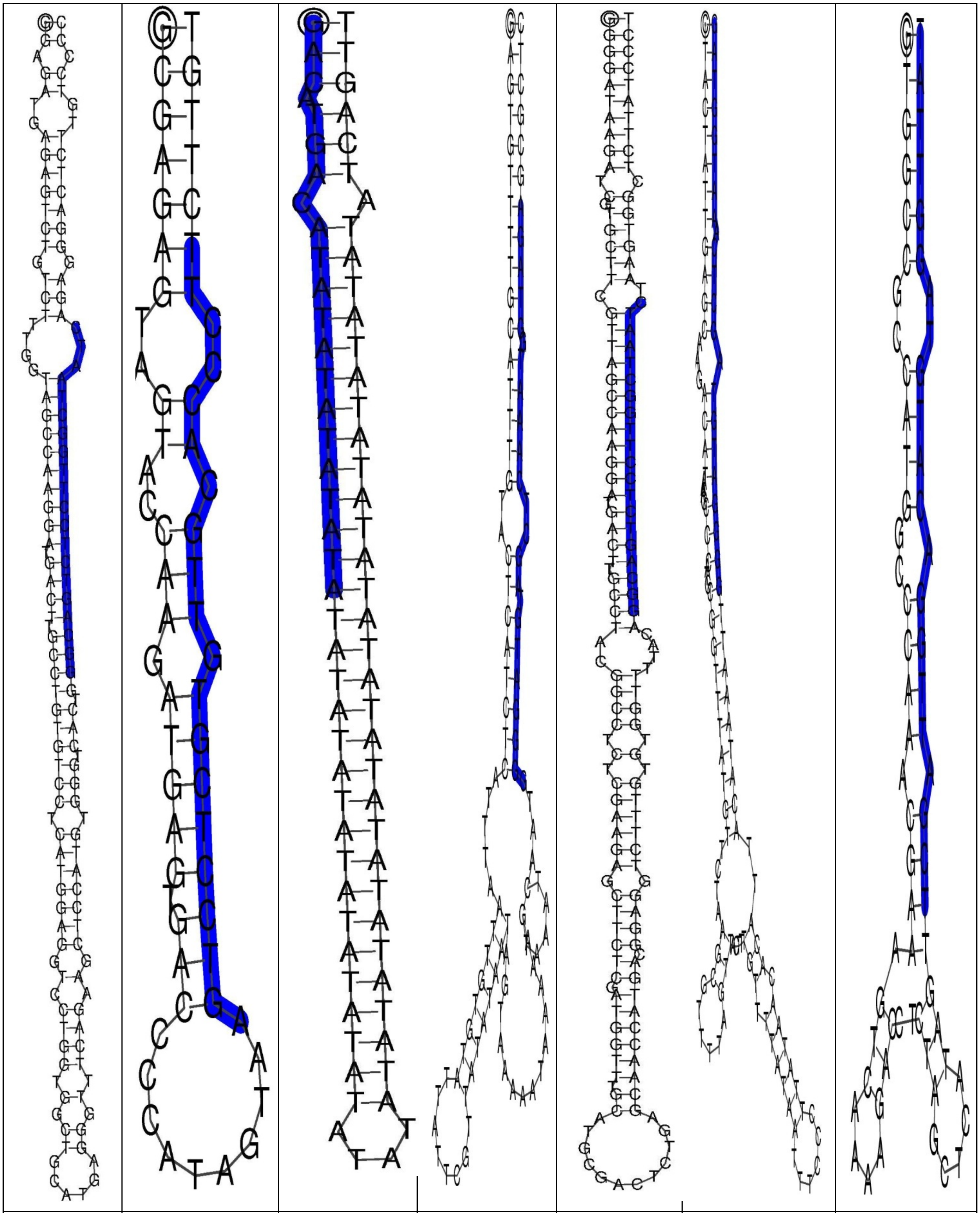

miRNA43	miRNA44	miRNA45	miRNA46	miRNA47	miRNA48	miRNA49

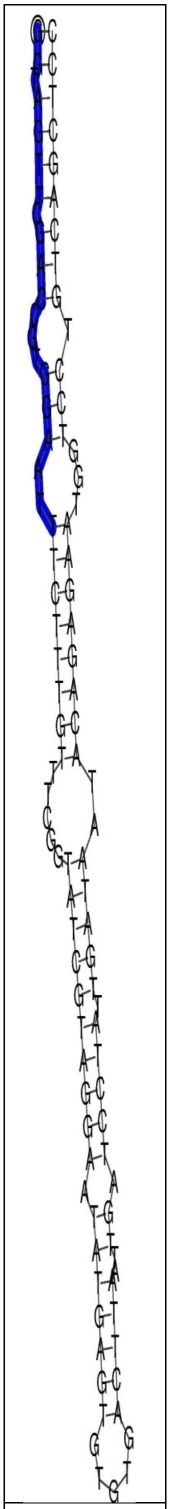

miRNA50
